# Imidazo[2,1-*b*][1,3,4]thiadiazol-2-yl]-1*H*-pyrrolo[2,3-*b*]pyridines as Inhibitors of *Staphylococcus aureus* Biofilm Formation

**DOI:** 10.3390/antibiotics15060598

**Published:** 2026-06-12

**Authors:** Domenico Schillaci, Fabiana Plescia, Fabio Scianò, Federica Parisi, Valentina Catania, Barbara Manachini, Serena Indelicato, Tania Ciaglia, Anna Di Dio, Stella Cascioferro

**Affiliations:** 1 Department of Biological, Chemical, and Pharmaceutical Sciences and Technologies (STEBICEF), University of Palermo, Via Archirafi 32, 90123 Palermo, Italy; domenico.schillaci@unipa.it (D.S.); fabiana.plescia@unipa.it (F.P.); fabio.sciano@live.it (F.S.); federica.parisi02@unipa.it (F.P.); serena.indelicato@unipa.it (S.I.); 2 Department of Earth and Marine Sciences (DiSTeM), University of Palermo, Viale delle Scienze Ed. 16, 90128 Palermo, Italy; valentina.catania@unipa.it; 3 Department of Agricultural, Food and Forest Sciences (SAAF), University of Palermo, Viale delle Scienze 13, 90128 Palermo, Italy; barbara.manachini@unipa.it; 4 Department of Pharmacy, University of Salerno, Via G. Paolo II 132, Fisciano, 84084 Salerno, Italy; tciaglia@unisa.it (T.C.); adidio6@studenti.unisa.it (A.D.D.)

**Keywords:** imidazothiadiazoles, antibiotic resistance, anti-virulence strategy, biofilm, staphylococcal infections, dispersal agents

## Abstract

**Background/Objectives**: Biofilm-related infections represent a major concern in clinical practice because of their poor responsiveness to standard antimicrobial treatments. Over the past decade, many efforts have been made to identify new compounds capable of inhibiting this particularly resistant form of bacterial life. Although several compounds have shown interesting anti-biofilm properties, none have reached the clinic. **Methods**: In the present work, we describe the synthesis and biological assessment as anti-biofilm agents, of a novel library of twenty-one imidazo[2,1-*b*][1,3,4]thiadiazol-2-yl]-1*H*-pyrrolo[2,3-*b*]pyridines. **Results**: The newly synthesized compounds were *in vitro* evaluated for their ability to prevent biofilm formation and to eradicate established biofilms in *Staphylococcus aureus* ATCC 25923 and *Pseudomonas aeruginosa* ATCC 15442. Additionally, the most potent compounds, **9g** and **9u**, were *in vivo* assayed for their safety profile and the anti-infective effect in a *Galleria mellonella* model. **Conclusions**: Derivatives **9g**, **9i**, **9n**, **9q** and **9u** proved to be potent inhibitors of *S. aureus* biofilm showing BIC_50_ values lower than 15 µg/mL. Noteworthy, the *in vivo* results revealed the promising protective properties of compound **9u** in the early stage of staphylococcal infection.

## 1. Introduction

The rise of bacterial strains resistant to common antibiotic treatments has posed a significant global health threat, with relevant economic and social impacts. The antibiotic resistance crisis is largely driven by the overprescription and improper use of antibiotics, combined with a drastic decrease in the development of new drugs [1]. Additionally, the situation has worsened in recent years, especially during the COVID-19 pandemic, when inappropriate and widespread antibiotic use led to a noticeable increase in multidrug-resistant (MDR) infections [2]. These infections, particularly in their chronic forms, have now become a leading cause of death across different age groups [3]. The rise in these diseases poses a significant threat to public health, necessitating urgent attention and innovative approaches to combat the growing crisis.

Among the different approaches proposed to fight the antibiotic resistance, the anti-virulence strategy is considered one of the most promising [4]. Compounds able to interfere with virulence mechanisms, without affecting microbial growth or viability, showed encouraging results in counteracting infections caused by MDR pathogens imposing very low selective pressure for the onset of new antibiotic resistance mechanisms [5]. Pathogenic bacterial genomes typically harbor a diverse range of virulence factors that collectively contribute to the evasion of the host’s immune defenses and the establishment of infectious disease [6]. These factors commonly include cell surface components that enable bacterial motility and adherence to host tissues, cell wall-degrading enzymes, toxins that damage host cells, immunosuppressive effectors, and molecules that facilitate nutrient acquisition or alter host cellular metabolism [7]. Based on their mode of interaction with host cells, virulence factors are categorized as either associated with the bacterial cell membrane or secreted into the extracellular environment.

A crucial weapon that most pathogens possess is the ability to grow as microbial communities known as biofilm [8]. Different key virulence factors, which promote adhesion, biofilm maturation, and immune evasion, are involved in the process of the biofilm formation. Bacteria inside biofilms result 1000 times more resistant than their planktonic counterparts [9]. There are many factors which can be held responsible for this resistance; some of these are related to the presence of the matrix which enhances bacterial adhesion to host surfaces, improves interbacterial communication, and provides an ideal environment for horizontal gene transfer, responsible for the antibiotic resistance development [10]. Additionally, bacteria inside biofilm preserve all the resistance mechanisms developed by planktonic forms, including enzymatic resistance, chemical modification of the antibiotic target and expression of efflux pumps. For all these reasons biofilm is a bacterial form of growth responsible for severe and chronic infections, which are very difficult to treat with conventional antibiotics [11].

**Figure 1:**
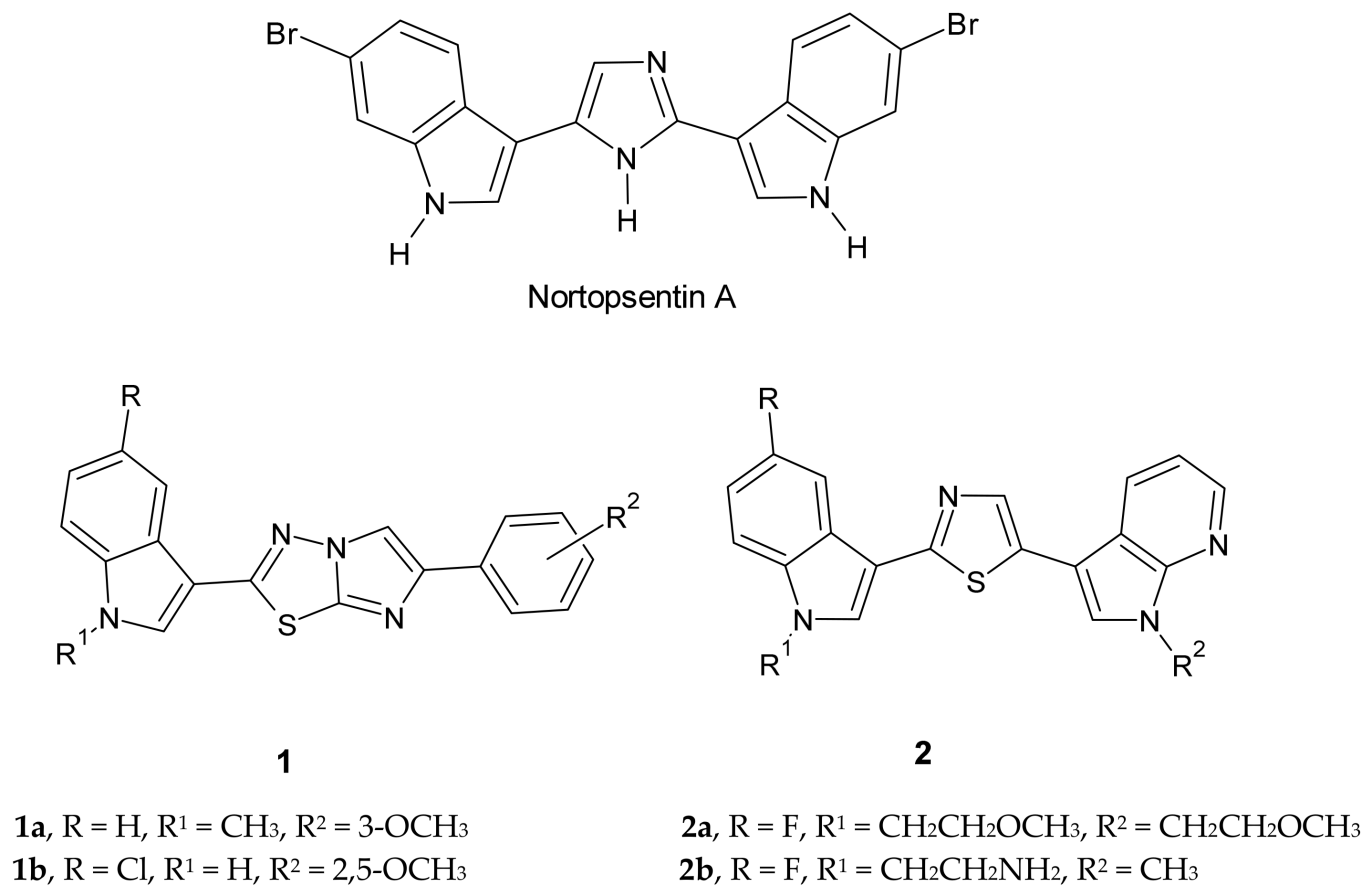
Chemical structure of the marine alkaloid nortopsentin A and synthetic anti-biofilm derivatives **1a**,**b** and **2a**,**b**.

Many efforts have been made in recent years to develop new strategies able to fight these resistant communities of pathogens, and different promising anti-biofilm agents have been identified, but unfortunately none have reached the clinic [12,13,14,15,16]. The absence of approved anti-biofilm therapies, combined with the growing incidence of chronic biofilm-associated nosocomial infections, underscores the critical importance of the research in this field [17,18]. In recent years, structural modification of the marine alkaloid nortopsentin (Figure 1) has allowed the discovery of potent anti-virulence derivatives that act by interfering with the initial stages of biofilm formation [19]. Among the different series of nortopsentin analogues synthesized, derivatives belonging to the classes **1** and **2** (Figure 1), in which the imidazole centrale core of the natural product was replaced with a thiazole or imidazo[2,1-*b*][1,3,4]thiadiazole moiety, respectively, exhibited remarkable anti-biofilm efficacy [6,7]. The compounds of type **1** and **2** showed a potent effect in inhibiting the first step of the biofilm formation. Additionally, compounds **1** and **2** exhibited a marked selectivity towards Gram-positive pathogens, particularly against staphylococcal strains exhibiting, in some cases, IC_50_ values lower than 1 µg/mL.

SAR analysis highlighted a key role of the indole scaffold for the anti-biofilm activity of these classes of compounds. Its replacement with other heterocycles was found detrimental for the activity.

Moreover, the relevance of indole scaffold in the development of antimicrobial agents is mainly due to its role as intercellular signaling molecule [20]. Indole is, in fact, produced by more than 85 species of Gram-positive and Gram-negative pathogens, in which it is involved in the biofilm formation processes and virulence factor production [21]. On the other hand, the imidazo[2,1-*b*][1,3,4]thiadiazole moiety and the 7-azaindole ring have been widely recognized as a privileged scaffolds for obtaining molecules with a broad spectrum of biological properties, including antibacterial and anti-biofilm activities [18,22].

Among the anti-biofilm compounds of type **1** and **2**, the derivatives **1a**,**b** and **2a**,**b** showed potent anti-biofilm activity, with IC_50_ values in the low micromolar range, exhibiting marked selectivity towards the Gram-positive tested pathogens *S. aureus* ATCC 25923, *S. aureus* ATCC 6538 and *S. epidermidis* ATCC 12228 [18].

Considering the urgent need to identify new compounds able of counteracting the phenomenon of antibiotic resistance and, on the basis of the promising anti-virulence activities found for the compounds of type **1** and **2**, we decided to synthesize a new series of anti-biofilm agents by combining the imidazothiadiazole central core of **1** with the 7-azaindole moiety of **2** in order to evaluate whether the simultaneous presence of both groups can lead to an increase in anti-biofilm properties. Furthermore, given the well-established importance of the indole nucleus for anti-biofilm activity, the effect of its replacement with the bioisostere 7-azaindole was evaluated.

## 2. Results and Discussion

### 2.1. Chemistry

**Scheme 1:**
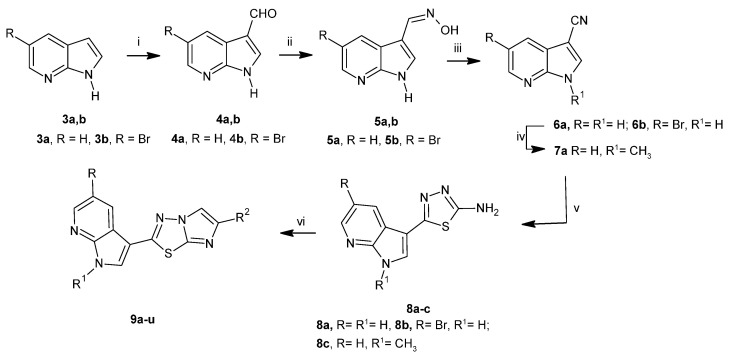
Synthesis of the new series of 3-(6-phenyl-imidazo[2,1-*b*][1,3,4]thiadiazol-2-yl)-1*H*-pyrrolo[2,3-*b*]pyridines **9a**–**u**. Reagents and conditions: (i) hexamethylenetetramine hydrochloride, acetic acid/water solution (1:2), 120 °C, 2 h (85–88%); (ii) hydroxylamine hydrochloride, EtOH/H_2_O (2:1), Na_2_CO_3_, 95 °C, 2 h (90–95%); (iii) acetic anhydride, 130 °C, 6 h, then reflux Methanol/NaOH 1 M solution, 2 h (80–85%); (iv) toluene, CH_3_I, potassium *t*-butoxide (t-BuOK), tris [2-(2-methoxyethoxy) ethyl] amine (TDA-1), r.t. (85%); (v) trifluoracetic acid, thiosemicarbazide, 60 °C, 3 h (95–98%); (vi) anhydrous ethanol, suitable α-bromoacetyl derivatives, reflux, 24 h (41–60%).

A new series of twenty-one 3-(6-phenyl-imidazo[2,1-*b*][1,3,4]thiadiazol-2-yl)-1*H*-pyrrolo[2,3-*b*]pyridine **9a**–**u** (Table 1) was successfully prepared following the synthetic procedure reported in Scheme 1.

In particular, the reaction of the commercial 7-azaindole **3** with hexamethylenetetramine hydrochloride in acetic acid/water solution (1:2), gave the 3-carbaldheyde analog **4** in good yield (85–88%) [23], which was in turn reacted with hydroxylamine hydrochloride to obtain oxime derivative **5** in excellent yield (90%). The 3-carbonitrile intermediate **7** was obtained by heating derivative **5** in acetic anhydride [23]. The methylation reaction to achieve compound **7** was conducted with iodomethane in presence of potassium *t*-butoxide as base and TDA-1 as a phase transfer catalyst. In this transformation, potassium tert-butoxide acts as a strong base, deprotonating the pyrrolic NH to generate the corresponding nitrogen anion, which is significantly more nucleophilic. Subsequently, methyl iodide serves as the alkylating agent, leading to SN2 methylation at nitrogen and formation of the N-methylated product. The phase-transfer catalytic effect of tris[2-(2-methoxyethoxy)ethyl]amine (TDA-1) facilitates the reaction in the organic medium, enhancing the efficiency of alkylation under mild conditions. The key intermediates **8a**–**c** were prepared by reaction of carbonitriles **6** and **7** [24,25], with thiosemicarbazide in trifluoroacetic acid at 60 °C for 3 h (yield 95–98%), specifically, the 2-aminothiadiazoles **8a**,**b** were obtained from cyano derivatives **6a**,**b**, whereas the methylated analogue **8c** was prepared from **7a** [26]. Finally, the reaction between the appropriate 5-(1*H*-pyrrolo[2,3-*b*]pyridin-3-yl)-1,3,4-thiadiazol-2-amine intermediates **8a**–**c** and the corresponding α-bromoacetyl derivatives, performed in anhydrous ethanol under reflux for 24 h, afforded the desired 3-(6-phenyl-imidazo[2,1-*b*][1,3,4]thiadiazol-2-yl)-1H-pyrrolo[2,3-*b*]pyridine analogues **9a**–**u** (yields 41–60%). In particular, compounds **9a**–**e** were obtained from intermediate **8a**, while methylated derivatives **9f**–**n** were synthesized starting from intermediate **8c**. Finally, compounds **9o**–**u** were prepared from intermediate **8b**.

### 2.2. Biology

#### 2.2.1. Antibacterial Activity

The newly synthesized compounds **9a**–**u** were initially tested for their *in vitro* antibacterial activity against the planktonic form of the Gram-positive pathogen *S. aureus* ATCC 25923 and the Gram-negative *P. aeruginosa* ATCC 1544. All the tested compounds elicited minimum inhibitory concentration (MIC) values exceeding 200 μg/mL, indicating no antibacterial activity. The lack of antibacterial activity (high MIC values) may seem unfavorable; however, it can be desirable when the aim is the identification of compounds with anti-virulence properties.

Anti-virulence agents, in fact, are defined as compounds capable of suppressing key bacterial virulence factors without affecting pathogen growth or viability [4]. Accordingly, the newly synthesized derivatives may function by disarming pathogens rather than exerting bactericidal effects. The anti-virulence strategy is considered a promising approach to counteract antibiotic resistance, since it imposes limited selective pressure in promoting the onset of new resistance mechanisms [27]. Among the numerous virulence attributes exploited by pathogens, biofilm formation stands out as one of the most significant, as it greatly enhances microbial resistance to conventional antibiotic treatments and host defenses and it is responsible for serious chronic infections especially in a hospital environment [28]. The new compounds were therefore tested to evaluate their anti-biofilm activity both by inhibiting its formation and by disrupting mature biofilm.

#### 2.2.2. Inhibition of Biofilm Formation

The new series of compounds **9a**–**u** was subsequently *in vitro* assayed at a concentration below the highest tested concentration in the MIC assay (200 µg/mL), corresponding to 100 µml, for evaluating their ability to inhibit biofilm formation against the tested strains, *S. aureus* ATCC 25923 and *P. aeruginosa* ATCC 15442 (Table 2). The new derivatives **9** showed a marked selectivity against the Gram-positive strain *S. aureus* ATCC 25923, proving to be effective in a dose-dependent manner.

Since the imidazothiadiazoles **9a**–**u** exhibited a significant inhibition of *S. aureus* biofilm formation at 100 µg/mL, eliciting percentages of inhibition ranging from 43 to 78%, the new derivatives were further tested at four lower concentrations (50, 25, 12.5 and 6.2 µg/mL). Percentages of inhibition greater than 50% was found up to concentration of 12.5 μg/mL for the two derivatives **9g** and **9u**.

At 100 µg/mL, most of the tested compounds exhibited low anti-biofilm activity against the Gram-negative pathogen *P. aeruginosa* ATCC 15442, with inhibition values below 50%, ranging from 39 to 12%. No activity was observed at 50 μg/mL, except for the derivatives **9g**, **9j**, **9n**, **9o** and **9r** with a percentage of inhibition, ranging from 22% to 14%.

For compounds producing more than 50% inhibition at 100 µg/mL, the BIC_50_ values were determined as the concentration causing a 50% reduction in biofilm formation compared with the untreated control (Table 3). Among the new compounds, derivatives **9g** and **9u** showed the highest potency against *S. aureus* ATCC 25923, with BIC_50_ values lower than 10 µg/mL, precisely 9.2 and 8.3 µg/mL, respectively. Moderate anti-biofilm activity (BIC_50_ values 10–20 µg/mL) was also observed for **9d**, **9i**, **9n**, **9o**, and **9q**, which exhibited BIC_50_ values ranging from 12.5 to 16.5 µg/mL.

Based on the evaluation of the obtained results, some considerations regarding the structure–activity relationship (SAR) of derivatives **9a**–**u** can be proposed. In particular, among derivatives bearing an unsubstituted azaindole moiety at position 5, the presence of a methyl group at R1 appears to be beneficial for anti-biofilm activity, as highlighted by compounds **9g**, **9i**, and **9n**. Regarding substitutions at position 6 of the imidazothiadiazole nucleus, the introduction of a 2,5-dimethoxyphenyl moiety (as in **9g** and **9q**) proved especially advantageous, suggesting a positive contribution of electron-donating aromatic substituents in this region. Similarly, the incorporation of a 3-thiophenyl group (**9u**) was associated with improved activity, further supporting the role of heteroaromatic substitution in enhancing the anti-biofilm profile of this scaffold.

Interestingly, additional selectivity trends also emerged from the biological evaluation. In particular, the presence of a bromine atom at position 5 of the indole ring, together with the introduction of either a 3-thiophenyl or a 2,5-dimethoxyphenyl substituent at position 6 of the imidazothiadiazole core, appeared to enhance selectivity toward Gram-positive strains. This observation may suggest that both the increased lipophilic character and the electronic properties conferred by these substituents contribute to a more favorable interaction with structural components specific to Gram-positive bacteria, although the precise molecular determinants underlying this selectivity still remain to be clarified.

#### 2.2.3. SEM Analysis of Biofilm Formation After Treatment with Compounds **9g** and **9u**

**Figure 2:**
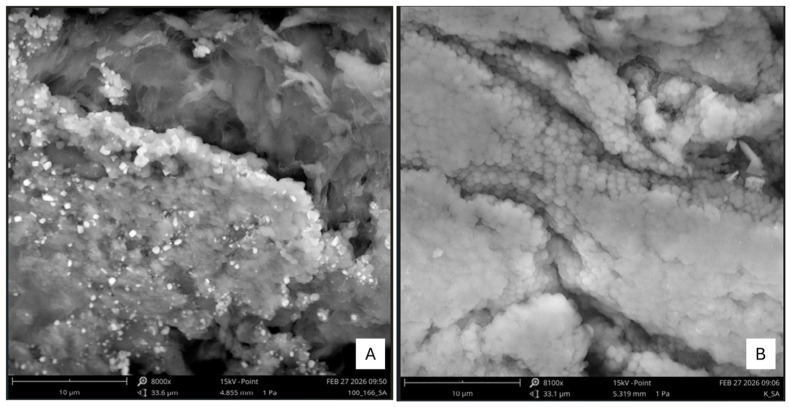
SEM images showing *S. aureus* ATCC 25923 biofilm after 24 h of treatment with **9g** at concentration of 100 µg/mL (**A**), and non-treated control (**B**). Bars: 10 µm.

**Figure 3:**
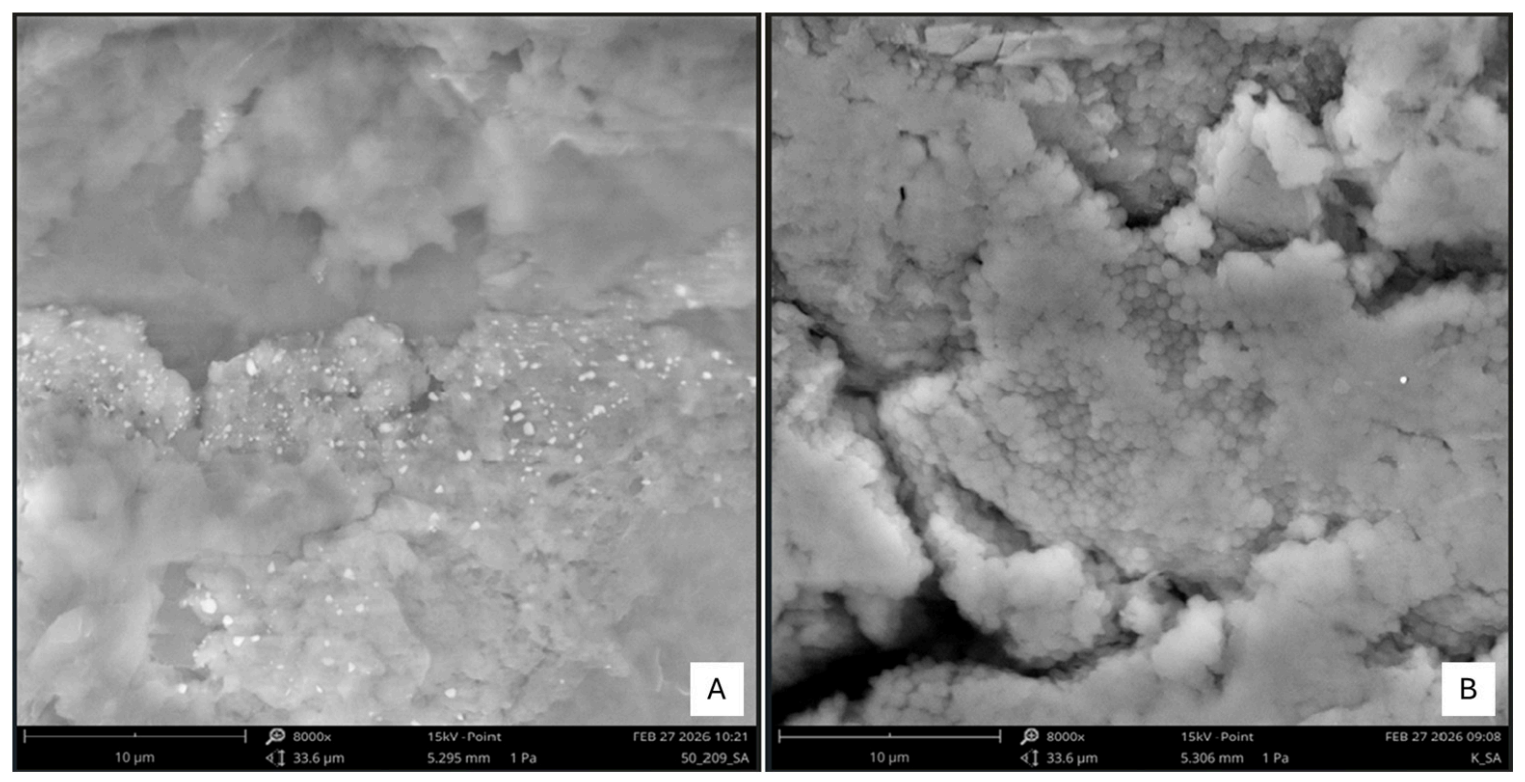
SEM images showing *S. aureus* ATCC 25923 biofilm after 24 h of treatment with **9u** at concentration of 100 µg/mL (**A**), and non-treated control (**B**). Bars: 10 µm.

Scanning electron microscopy (SEM) was used to evaluate changes in biofilm formation of *S. aureus* ATCC 25923 after treatment with compounds **9g** and **9u.** The images obtained showed that, at the highest concentrations tested, both compounds **9g** and **9u** caused a clear reduction in biofilm formation of *S. aureus* (Figure 2 and Figure 3).

In particular, the samples (Figure 2A and Figure 3A) showed a markedly disrupted biofilm architecture, characterized by a lower cell density, reduced extracellular matrix, and the absence of the thick, multilayered and compact structure observed in the untreated growth control (Figure 2B and Figure 3B), which displayed a dense and well-organized biofilm. These observations support the anti-biofilm activity of both compounds, in agreement with the biofilm assay results.

#### 2.2.4. Dispersal Activity Against Pre-Formed Biofilm

Compounds **9–u** were further assayed in order to evaluate also their dispersal activity against staphylococcal mature biofilm. A preliminary screening at three different concentrations (200 μg/mL, 100 μg/mL and 50 μg/mL) was performed on 24 h preformed biofilm of *S. aureus* ATCC 25923 (Table 4). Compounds **9h**, **9i** and **9k** showed the highest anti-biofilm activity against 24 h preformed *S. aureus* ATCC 25923 biofilms, with substantial inhibition even at 50 µg/mL. Derivatives **9i** and **9q** displayed moderate but consistent inhibition across all tested concentrations (45–50%), while **9j**, **9n**, and **9o** were less active, with inhibition values ranging from 20% to 40%. The remaining compounds were inactive against preformed biofilm, displaying non-significant inhibitory effects (<10%). The apparent discrepancy between the inhibition of the biofilm formation and dispersal activities of compounds **9**, can be rationalized by considering that these two processes involve distinct biological targets.

For the most potent imidazothiadiazoles **9h**, **9i** and **9k**, the BIC_50_ values were evaluated and are reported in Table 5. Compound **9h**, bearing the imidazothiadiazole scaffold substituted in position 2 with a 7-azaindole ring and in position 6 with the 2,5-dimethoxyphenyl, elicited the highest potency in disrupting biofilm architecture, exhibiting a BIC_50_ value of 39.7 μg/mL. Noteworthy, compound **9h** showed a dual action on the *S. aureus* biofilm, being capable of both preventing its formation and disrupting its mature structure. For compound **9q**, the minor differences observed between 100 and 200 μg/mL were not considered indicative of a true concentration-dependent decrease in activity, but rather reflect the intrinsic variability typically associated with preformed biofilm assays.

The selective anti-biofilm activity displayed by the newly synthesized imidazothiadiazole derivatives toward Gram-positive strains may reflect interference with molecular pathways that are particularly relevant for biofilm establishment and maturation in these organisms. In this regard, several potential targets can be hypothesized. Gram-positive biofilm formation, especially in staphylococcal species, strongly depends on processes such as teichoic acid biosynthesis and cell-wall organization, peptide-mediated quorum sensing systems (e.g., Agr), and the production of polysaccharide intercellular adhesin (PIA/PNAG), all of which are absent or substantially different in Gram-negative bacteria. Therefore, the preferential activity observed against Gram-positive biofilms could arise from selective perturbation of one or more of these pathways.

Importantly, the identification of the exact molecular mechanism remains challenging, as biofilm formation is a highly multifactorial process involving interconnected regulatory and structural pathways. Additionally, these compounds may not act through a single target, but rather through the simultaneous modulation of multiple processes contributing to biofilm development and stability. Such a multitarget mode of action could also explain the anti-biofilm activity observed in the absence of strong antibacterial effects on planktonic growth.

Among the potential Gram-positive-associated targets, Sortase A (SrtA) initially appeared particularly attractive due to its central role in anchoring surface adhesins involved in staphylococcal biofilm formation. However, preliminary enzymatic studies did not reveal any inhibitory activity of the imidazothiadiazole derivatives against SrtA, allowing us to reasonably exclude this membrane transpeptidase as a primary molecular target of the new series.

Further mechanistic investigations, including transcriptomic, proteomic, and genetic approaches, will therefore be necessary to clarify the molecular determinants underlying the selective anti-biofilm activity of these imidazothiadiazole derivatives and to establish whether multiple pathways are concomitantly involved.

### 2.3. In Vivo Anti-Infective Evaluation of ***9g*** and ***9u***

**Figure 4:**
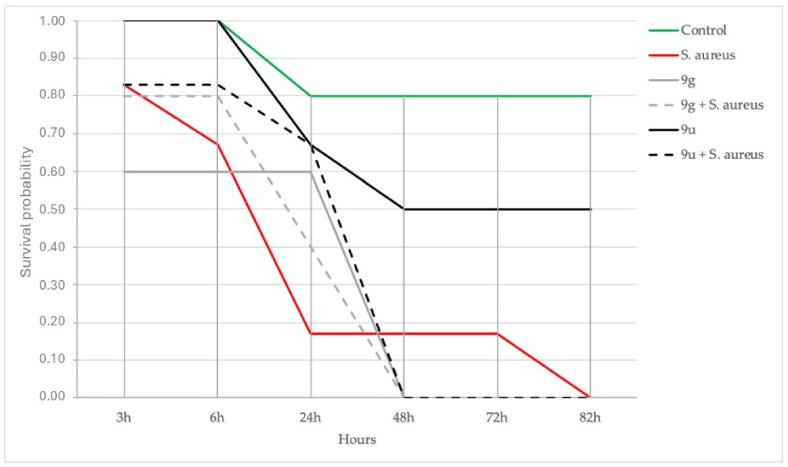
Survivorship of *G. mellonella* larvae infected with *S. aureus* and larvae infected *S. aureus* (2.8 × 10^4^ CFU/mL) and injected with a single dose (1 mg/kg) of the compound **9g** and **9u** separately. Values were determined by the Kaplan–Meier method and compared using the log-rank test (χ2 14.19, *p* = 0.0145).

The potential protective effects of compounds **9g** and **9u** were evaluated in an *in vivo* infection model using *Galleria mellonella* L. (Lepidoptera: Pyralidae) larvae [29]. Larval survival following co-inoculation with *S. aureus* and the tested compounds was compared with that of larvae infected with the bacterial strain alone. The use of *G. mellonella* larvae as an *in vivo* model in preclinical research has gained increasing attention in recent years due to several advantageous features [30]. Notably, these larvae share important similarities with conventional vertebrate animal models, including the presence of both humoral and cellular components of the innate immune system. Furthermore, specific biological characteristics of *G. mellonella*, such as their relatively large larval size, ease of maintenance, and ability to survive at temperatures up to 37 °C, contribute significantly to their suitability as an alternative model organism to mammals for *in vivo* studies. Owing to these advantages, *G. mellonella* represents a practical, rapid, and cost-effective system for the preliminary *in vivo* evaluation of compound toxicity and therapeutic efficacy [31]. *S. aureus* was selected as the test pathogen due to its clinical relevance, particularly in relation to the widespread occurrence of antibiotic-resistant strains. Moreover, this bacterium is known to act as a pathogen not only in humans but also in insects, making it suitable for severe infection studies using *G. mellonella* larvae. The long rank test showed significant overall differences between survival curves (χ2 = 14.19, *p* = 0.0145) (Figure 4). Pairwise comparisons (uncorrected *p* and False Discovery Rate, FDR, at 0.05) showed significant differences for the following pairs: Control vs. *S. aureus* (*p* = 0.0084 and FDR = 0.104); Control vs. **9g** (*p* = 0.0305 and FDR = 0.104); Control vs. **9g** + *S. aureus* (*p* = 0.0203 and FDR = 0.104); Control vs. **9u** + *S. aureus* (*p* = 0.0290 (FDR = 0.104); **9u** vs. *S. aureus* (*p* = 0.0348 (FDR = 0.104). However, considering the Fixed Time Comparisons (vs. Control)—Fisher exact (*p* two-sided) it is noted that at 24 h the treatments with *S. aureus* have a significantly different trend compared to all the other treatments (*p* = 0.008).

Larvae infected solely with *S. aureus* showed a rapid decrease in survival, which declined from 67% at 6 h to 17% at 24 h, with complete mortality occurring by 82 h. These findings confirm the high virulence of the pathogen in the *G. mellonella* infection model.

Both tested compounds showed a protective effect against *S. aureus* infection during the first 6 h, which was more pronounced for derivative **9u**.

Compound **9u** exhibited more marked protective effect especially during the early stages of infection, at the concentration tested in this study.

### 2.4. Toxicity Evaluation and Risk Analysis in In Vivo Model

The toxicity of the most active derivative, compounds **9g** and **9u**, was assessed in an *in vivo* model using *G. mellonella* larvae. A single dose of 1 mg/kg, dissolved in NaCl 0.9% (*v*/*v*), was administered to groups of 6 larvae. The effects were observed at 6, 24, 48, 72 and 82 h post-treatment. The toxicity assessment was based on the evaluation of the percentage of larval death. By monitoring the mortality percentage, the potential adverse effects of compounds **9g** and **9u** were evaluated in this model. The comparison of the mortality percentages between the group of larvae treated with compounds **9g** and **9u** and the control group (which received injections of the solvent only) indicates a certain level of toxicity of the compounds at the examined dose and regimen. This finding suggests that compound **9u** is better tolerated than **9g**. Moreover, the risk analysis (Table 6) indicates that treatment with **9g** is associated with a markedly unfavorable profile relative to the control group. A pronounced early peak in new infection events is observed within the 0–3 h interval, followed by a substantial surge in infection incidence between 24 and 48 h. In contrast to **9g**, the risk profile of **9u** more closely resembles that of the control group during the initial hours of observation, with only moderate increases in risk emerging between 6 and 48 h. This trend aligns well with the survival curves, where **9u** delays, but does not prevent, the occurrence of infection events when compared with infected conditions. When compared to the negative control, however, **9u** introduces a modest degree of additional risk (Table 6), a pattern that may be expected if the compound possesses some level of biological activity rather than being completely inert.

## 3. Materials and Methods

### 3.1. Chemistry

All reagents and solvents were obtained from commercial suppliers (Merck, Darmstadt, Germany; VWR International, Radnor, PA, USA; Alfa Aesar, Ward Hill, MA, USA; and Acros Organics, Geel, Belgium) and used as received, without further purification. Melting points were determined using a Büchi-Tottoly capillary melting point apparatus (Büchi, Cornaredo, Italy) and are reported uncorrected. ^1^H and ^13^C NMR spectra were recorded at 300 and 50 MHz, respectively, in deuterated dimethyl sulfoxide (DMSO) using a Bruker Avance II 300 MHz spectrometer (Bruker, Milan, Italy). Flash column chromatography was performed using silica gel 230–400 mesh (Merck) or with a Büchi Sepacor automated chromatography system equipped with prepacked cartridges. Elemental analyses for carbon, hydrogen, and nitrogen (C, H, N) were performed on a VARIO EL III elemental analyzer (Elementar Analysensysteme GmbH, Langenselbold, Germany) and were within ±0.4% of the calculated theoretical values. LC–MS and MS/MS analyses were performed on a Q Exactive 120 mass spectrometer (Thermo Fisher Scientific, Waltham, MA, USA) coupled to an LC system equipped with a Hypersil Gold column (Thermo Fisher Scientific) for the assessment of the chromatographic purity of the compounds.

#### 3.1.1. Synthesis of 1H-Pyrrolo[2,3-*b*]pyridine-3-carbaldehyde **4a**,**b**

To a solution of 8.4 mmol of 7-azaindole **3a**,**b** in acetic acid/water solution (1:2, 8.3 mL) hexamethylenetetramine hydrochloride (1.3 g) was added. The resulting mixture was heated at 120 °C for 6 h. After cooling to room temperature the mixture was poured into ice and filtered to afford the final compounds as white solids in good yield (85–88%). Analytical and spectroscopic data for derivatives **3** are in accordance with those reported in the literature [23].

#### 3.1.2. Synthesis of 1H-Pyrrolo[2,3-*b*]pyridine-3-carbaldehyde oxime **5a**,**b**

A solution of 6.8 mmol of 1*H*-pyrrolo[2,3-*b*]pyridine-3-carbaldehyde **4** in anhydrous ethanol (60 mL) was treated with 1.68 g of hydroxylamine hydrochloride and a solution of 1.30 g of sodium carbonate 28 mL of water. The mixture was refluxed for 2 h, and then the solvent was reduced under pressure and the obtained solid was filtered off and washed with cold water. The oxime was obtained in excellent yield (90–95%) and used for the next reaction without further purification. Analytical and spectroscopic data for the derivatives **3** are in accordance with those reported in the literature [23].

#### 3.1.3. Synthesis of 1H-Pyrrolo[2,3-*b*]pyridine-3-carbonitrile **6a**,**b**

A mixture of 1*H*-pyrrolo[2,3-*b*]pyridine-3-carbaldehyde oxime **5** (6.21 mmol) and acetic anhydride (53 mmol) was heated under reflux for 4 h. The reaction mixture was cooled to room temperature and poured into ice-cold water. The precipitate obtained was filtered and washed with ice-cold water. The white solid was suspended in water (20 mL) and the aqueous suspension was heated under reflux for 3 h. The reaction mixture was cooled to room temperature and poured into ice-cold water. The white solid obtained was filtered, washed with cold water and used for the next reaction without further purification (yield: 80–85%). Analytical and spectroscopic data for the derivatives **3** are in accordance with those reported in the literature [24].

#### 3.1.4. Synthesis of 1-Methyl-pyrrolo[2,3-*b*]pyridine-3-carbonitrile **7a**

To a cold solution (0 °C) of 1*H*-pyrrolo[2,3-*b*]pyridine-3-carbonitrile **6** (2.5 mmol) in anhydrous toluene (25 mL), tBuOK (0.38 g, 3.4 mmol) and TDA-1 (2 drops) were added. The reaction mixture was stirred at room temperature for 3 h and then methyl iodide (2.5 mmol, 0.2 mL) was added at 0 °C. After 1 h the solvent was evaporated under reduced pressure. The obtained residue was treated with water, extracted with DCM (3 × 20 mL), dried over anhydrous Na_2_SO_4_, evaporated and purified by column chromatography using DCM/ethyl acetate (9/1) as eluent, to give the desired product as white solid (yield: 85%). Analytical and spectroscopic data for the derivative **7a** are in accordance with those reported in the literature [25].

#### 3.1.5. Synthesis of 5-(1H-Pyrrolo[2,3-*b*]pyridin-3-yl)-[1,3,4]thiadiazol-2-ylamines **8a**–**c**

Equimolar amounts of the suitable 1*H*-pyrrolo[2,3-*b*]pyridine-3-carbonitrile **6**, **7** (5 mmol) and thiosemicarbazide (5 mmol) were heated under stirring at 60 °C for 3 h in trifluoroacetic acid (5 mL). After cooling, the mixture was poured into ice and treated with NaHCO_3_ saturated solution up to pH 7. The product, obtained as white solid, was filtered off, washed with water, cyclohexane and diethyl ether to give thiadiazolamines **8** in excellent yields (95–98%). Analytical and spectroscopic data for the derivatives **8** are in accordance with those reported in the literature [26].

#### 3.1.6. General Procedure for the Synthesis of 3-(6-Phenyl-imidazo[2,1-b][1,3,4]thiadiazol-2-yl)-1H-pyrrolo[2,3-*b*]pyridines **9a**–**u**

A mixture of equimolar amounts of the amines **8** (0.92 mmol) and the suitable α-bromoacetyl derivative (0.92 mmol) was refluxed under stirring anhydrous ethanol (30 mL) for 24 h. After cooling at room temperature the desired product **9** was filtered off and washed with cold ethanol. The chromatographic purity of compounds **9g** and **9u** was confirmed by LC–MS and MS/MS analyses using a Q Exactive 120 mass spectrometer (Thermo Fisher Scientific) equipped with a Hypersil Gold column (Thermo Fisher Scientific). Gradient elution was performed using solvent A consisting of H_2_O containing 0.1% formic acid and solvent B consisting of ACN/MeOH (50:50, *v*/*v*) containing 0.1% formic acid.

The electrospray ionization (ESI) source parameters were set as follows: positive ion mode, capillary voltage 3.5 kV, sheath gas flow 60 arb units, auxiliary gas flow 15 arb units, and Orbitrap resolution 60,000. MS data were acquired in full-scan mode (from 300 to 450 *m*/*z* range). MS/MS spectra were obtained using higher-energy collisional dissociation (HCD) at collision energies of 20, 40, and 60%, with an Orbitrap resolution of 15,000 and automatic scan range selection. The precursor ions selected for MS/MS spectra were *m*/*z* 392.1175 for compound **9g** and *m*/*z* 401.9477 for compound **9u**. HPLC traces (Figures S1 and S2 SI) confirm >95% purity for the lead compounds used in the G. mellonella studies (named as **9g** and **9u**). ^13^C NMR spectra were not acquired for compounds **9h**,**n** due to their poor solubility in DMSO.

##### 3-[6-(3-Methoxy-phenyl)-imidazo[2,1-b][1,3,4]thiadiazol-2-yl]-1H-pyrrolo[2,3-*b*]pyridine **9a**

Yield: 46%; pale yellow powder; m.p.: 244 °C; IR (cm^−1^): 3290 (NH) ^1^H NMR (300 MHz, DMSO-*d*_6_) δ 3.82 (3H, s, CH_3_), 6.85 (1H, dd, *J* = 8.4, 2.6 Hz, Ar), 7.38–7.29 (2H, m, Ar), 7.46 (2H, s, Ar), 8.36–8.56 (3H, m, Ar), 8.73 (1H, s, Ar), 12.68 (1H, s, NH). ^13^C NMR (50 MHz, DMSO-*d*_6_) δ 55.5, 105.9, 110.3, 111.3, 113.3, 116.7, 117.4, 118.2, 129.2, 130.2, 135.9, 143.5, 145.2, 145.3, 149.3, 157.0, 157.1, 160.1. Anal. Calculated for C_18_H_13_N_5_OS (MW: 347.39): C, 62.23%; H, 3.77%; N, 20.16%. Found: C, 62.45%; H, 3.91%; N, 20.47%.

##### 3-[6-(4-Fluoro-phenyl)-imidazo[2,1-*b*][1,3,4]thiadiazol-2-yl]-1*H*-pyrrolo[2,3-*b*]pyridine **9b**

Yield: 50%; yellowish powder; m.p.: 243 °C; IR (cm^−1^): 3396 (NH) ^1^H NMR (300 MHz, DMSO-*d*_6_) δ 7.25 (2H, td, *J* = 3, 2, 1.8 Hz, Ar), 7.35–7.31 (1H, m, Ar), 7.91 (2H, td, *J* = 3, 3.4, 4.6 Hz, Ar), 8.40 (1H, dd, *J* = 4.7, 1.6 Hz, Ar), 8.48 (2H, dd, *J* = 8.0, 1.7 Hz, Ar), 8.67 (1H, s, Ar), 12.64 (1H, s, NH). ^13^C NMR (50 MHz, DMSO-*d*_6_) δ: 107.1, 110.8, 115.9, 116.4, 118.2, 126.8, 127.0 (x2), 129.3, 130.2, 134.3, 143.5, 144.5 (x2), 145.1, 149.2, 153.7.Anal. Calculated for C_17_H_10_FN_5_S (MW: 335.36): C, 60.89%; H, 3.01%; N, 20.88%. Found: C, 61.07%; H, 3.30%; N, 20.99%.

##### 3-(6-Thiophen-3-yl-imidazo[2,1-*b*][1,3,4]thiadiazol-2-yl)-1*H*-pyrrolo[2,3-*b*]pyridine **9c**

Yield: 42%; whitish powder; m.p.: 255 °C; IR (cm^−1^): 3298 (NH); ^1^H NMR (300 MHz, DMSO-*d*_6_) δ 7.22 (1H, t, *J* = 3.9 Hz, Ar), 7.33 (1H, dd, *J* = 7.9, 4.7 Hz, Ar), 7.54 (1H, dd, *J* = 5.0, 1.1 Hz, Ar), 7.61 (1H, dd, *J* = 5.0, 2.9 Hz, Ar), 7.77 (1H, dd, *J* = 2.9, 1.0 Hz, Ar), 8.41 (1H, dd, *J* = 4.7, 1.4 Hz, Ar), 8.52–8.47 (2H, m, Ar), 8.54 (1H, s, Ar), 12.66 (1H, s, NH). ^13^C NMR (50 MHz, DMSO-*d*_6_) δ: 105.9, 106.8, 110.7, 116.7, 118.2, 119.9, 126.0, 127.2, 129.3, 130.1, 136.4, 142.3, 145.2, 149.3, 156.9.Anal. Calculated for C_15_H_9_N_5_S_2_ (MW: 323.39): C, 55.71%; H, 2.81%; N, 21.66%. Found: C, 55.98%; H, 2.96%; N, 21.85%.

##### 3-(6-Pyridin-3-yl-imidazo[2,1-*b*][1,3,4]thiadiazol-2-yl)-1*H*-pyrrolo[2,3-*b*]pyridine **9d**

Yield: 41%; grey powder; m.p.: 267 °C; IR (cm^−1^): 3320 (NH);^1^H NMR (300 MHz, DMSO-*d*_6_) δ: 7.34 (1H, dd, *J* = 8.0, 4.7 Hz, Ar), 7.95 (1H, t, *J* = 6.9 Hz, Ar), 8.56–8.39 (3H, m, Ar), 8.75 (2H, dd, *J* = 14.3, 6.8 Hz, Ar), 8.99 (1H, d, *J* = 1.8 Hz, Ar), 9.25 (1H, s, Ar), 12.66 (1H, s, NH). ^13^C NMR (50 MHz, DMSO-*d*_6_) δ: 105.6, 113.7, 116.7, 118.3, 127.0, 129.3, 129.9, 130.8, 133.0, 138.7, 139.8, 139.9, 142.1, 145.0, 145.2, 149.2, 158.4. Anal. Calculated for C_16_H_10_N_6_S (MW: 318.36): C, 60.36%; H, 3.17%; N, 26.40%. Found: C, 60.65%; H, 3.35%; N, 26.69%.

##### 3-[6-(1,3-Thiazol-2-yl)imidazo[2,1-*b*][1,3,4]thiadiazol-2-yl]-1*H*-pyrrolo[2,3-*b*]pyridine **9e**

Yield: 58%; whitish powder; m.p.: 308 °C. ^1^H NMR (300 MHz, DMSO-*d*_6_) δ: 7.27–7.15 (3H, m, Ar-H), 8.00 (1H, d, *J* = 2.3 Hz, Ar), 8.32 (1H, dd, *J* = 4.7, 1.7 Hz, Ar), 8.44 (1H, dd, *J* = 7.9, 1.7 Hz, Ar), 8.49–8.54 (1H, m, Ar), 12.15 (1H, s, NH). ^13^C NMR (50 MHz, DMSO-*d*_6_) δ: 105.7, 106.8, 111.8, 116.7, 117.1, 117.3, 127.1, 129.6, 130.7, 143.9, 144.4, 149.1, 152.2, 166.5. Anal. Calculated for C_14_H_8_N_6_S_2_ (MW: 324.38): C, 51.84%; H, 2.49%; N, 25.91%. Found: C, 51.98%; H, 2.73%; N, 26.12%.

##### 1-Methyl-3-(6-phenyl-imidazo[2,1-*b*][1,3,4]thiadiazol-2-yl)-1*H*-pyrrol [2,3-*b*]pyridine **9f**

Yield: 44%; whitish powder; m.p.: 221 °C; ^1^H NMR (300 MHz, DMSO-*d*_6_) δ: 3.92 (3H, s, CH_3_), 7.28 (1H, t, *J* = 7.3 Hz, Ar), 7.40 (3H, dt, *J* = 7.9, 6.2 Hz, Ar), 7.89 (2H, d, *J* = 7.7 Hz, Ar), 8.48 (2H, t, *J* = 5.8 Hz, Ar), 8.54 (1H, s, Ar), 8.69 (1H, s, Ar). ^13^C NMR (50 MHz, DMSO) δ 31.9, 104.6, 111.0, 117.0, 118.4, 125.0, 127.6, 129.1, 129.5, 133.4, 134.4, 145.0, 145.4, 148.2, 156.7. Anal. Calculated for C_18_H_13_N_5_S (MW: 331.39): C, 65.24%; H, 3.95%; N, 21.13%.

##### 3-[6-(2,5-Dimethoxy-phenyl)-imidazo[2,1-*b*][1,3,4]thiadiazol-2-yl]-1-methyl-1*H*-pyrrolo[2,3-*b*]pyridine **9g**

Yield: 47%; yellowish powder; m.p.: 245 °C; ^1^H NMR (300 MHz, DMSO-*d*_6_) δ: 3.77 (3H, s, CH_3_), 3.90 (3H, s, CH_3_), 3.92 (3H, s, CH_3_), 6.87 (1H, dd, *J* = 8.9, 2.8 Hz, Ar), 7.03 (1H, t, *J* = 7.8 Hz, Ar), 7.38 (1H, dd, *J* = 7.7, 4.9 Hz, Ar), 8.52 (1H, d, *J* = 1.1 Hz, Ar), 8.47 (2H, d, *J* = 7.4 Hz, Ar), 8.58 (2H, s, Ar). ^13^C NMR (50 MHz, DMSO-*d*_6_) δ: 31.3, 55.8, 56.3, 103.8, 104.5, 113.8, 116.8, 117.0, 118.6, 122.9, 129.6, 133.6, 140.8, 143.9, 145.0, 149.0, 150.4, 151.5, 153.7, 156.0. Anal. Calculated for C_20_H_17_N_5_O_2_S (MW: 391.45): C,61.37%; H,4.38%; N,17.89%. Found: C,61.59%; H, 4.67%; N,18.06%.

##### 1-Methyl-3-(6-thiophen-3-yl-imidazo[2,1-*b*][1,3,4]thiadiazol-2-yl)-1*H*-pyrrolo[2,3-*b*]pyridine **9h**

Yield: 41%; light brown powder; m.p.: 270 °C; ^1^H NMR (300 MHz, DMSO-*d*_6_) δ: 3.92 (3H, s, CH_3_), 7.38 (1H, d, *J* = 2.5 Hz, Ar), 7.48–7.70 (2H, m, Ar), 7.79 (1H, d, *J* = 3.2 Hz, Ar), 8.36–8.65 (4H, m, Ar). Anal. Calculated for C_16_H_11_N_5_S_2_ (MW: 337.42): C, 56.95%; H, 3.29%); N, 20.76%. Found: C, 57.21%; H, 3.60%); N, 20.98%.

##### 1-Methyl-3-(6-pyridin-3-yl-imidazo[2,1-*b*][1,3,4]thiadiazol-2-yl)-1*H*-pyrrolo[2,3-*b*]pyridine **9i**

Yield: 60%; yellow powder; m.p.: 243 °C; ^1^H NMR (300 MHz, DMSO-*d*_6_) δ: 3.89 (3H, s, CH_3_), 7.43–7.31 (1H, m, Ar), 7.93 (1H, dd, *J* = 7.6, 4.5 Hz, Ar), 8.44 (2H, d, *J* = 5.1 Hz, Ar), 8.54 (1H, s, Ar), 8.71 (2H, d, *J* = 4.8 Hz, Ar), 8.96 (1H, s, Ar), 9.21 (1H, s, Ar). ^13^C NMR (50 MHz, DMSO-*d*_6_) δ 31.9, 104.3, 113.6, 116.8, 118.5, 124.9, 126.9, 129.5, 132.9, 133.9, 138.4, 139.8, 140.0, 142.2, 145.0, 148.2, 157.9. Anal. Calculated for C1_7_H_12_N_6_S (MW: 332.38): C, 61.43%; H, 3.64%; N, 25.28%. Found: C, 61.68%; H, 3.91%; N, 25.41%.

##### 3-[6-(4-Fluoro-phenyl)-imidazo[2,1-*b*][1,3,4]thiadiazol-2-yl]-1-methyl-1*H*-pyrrolo[2,3-*b*]pyridine **9j**

Yield: 63%; yellow powder; m.p.: 238 °C, ^1^H NMR (300 MHz, DMSO-*d*_6_) δ: 3.90 (3H, s, CH_3_), 7.43–7.19 (3H, m, Ar-H), 7.93–7.87 (1H, m, Ar-H), 8.03–7.95 (1H, m, Ar-H), 8.50–8.41 (2H, m, Ar-H), 8.54 (1H, d, *J* = 7.7 Hz, Ar-H), 8.66 (1H, s, Ar-H). ^13^C NMR (50 MHz, DMSO-*d*_6_) δ: 31.9, 104.3, 104.5, 110.9, 115.8, 116.1, 116.9, 118.5 (d, *J* = 9.5 Hz), 126.9 (d, *J* = 8.1 Hz), 128.5 (d, *J* = 8.1 Hz), 129.5, 130.9, 133.4, 133.8, 143.4, 144.4, 144.9, 148.2, 156.7, 157.7. Anal. Calculated for C_18_H_12_ FN_5_S (MW: 348.39): C, 61.88%; H, 3.46%; N, 20.05%. Found: C, 61.97%; H, 3.68%; N, 20.24%.

##### 3-[6-(2,3-Dihydro-benzo[1,4]dioxin-6-yl)-imidazo[2,1-*b*][1,3,4]thiadiazol-2-yl]-1-methyl-1*H*-pyrrolo[2,3-*b*]pyridine **9k**

Yield: 58%; white powder; m.p.: 257 °C; ^1^H NMR (300 MHz, DMSO-*d*_6_) δ: 3.94 (3H, s, CH_3_), 4.29 (4H, d, *J* = 8.0 Hz, CH_2_), 6.81–7.00 (1H, m, Ar), 7.25–7.63 (3H, m, Ar), 8.32–8.68 (4H, m, Ar). ^13^C NMR (50 MHz, DMSO-*d*_6_): δ 31.9, 64.6, 104.5, 110.3, 113.7, 117.0, 117.8, 118.3, 118.4, 119.7, 127.4, 129.5, 133.4, 143.4, 144.0, 144.7, 144.9, 148.2, 156.7. Anal. Calculated for C_20_H_15_N_5_O_2_S (MW: 389.43): C, 61.68%; H, 3.88%; N, 17.98%. Found: C, 61.81%; H, 3.97%; N, 18.12%.

##### 3-(6-Furan-2-yl-imidazo[2,1-*b*][1,3,4]thiadiazol-2-yl)-1-methyl-1*H*-pyrrolo[2,3-*b*]pyridine **9l**

Yield: 55%; brown powder; m.p.: 221 °C; ^1^H NMR (300 MHz, DMSO-*d*_6_) δ: 3.91 (3H, s, CH_3_), 6.59 (1H, dd, *J* = 3.2, 1.8 Hz, Ar), 6.70 (1H, d, *J* = 3.2 Hz, Ar), 7.37 (1H, dd, *J* = 7.8, 4.7 Hz, Ar), 7.70 (1H, d, *J* = 0.8 Hz, Ar), 8.40 (1H, s, Ar), 8.47 (2H, dd, *J* = 9.7, 2.1 Hz, Ar), 8.55 (1H, s, Ar). ^13^C NMR (50 MHz, DMSO-*d*_6_) δ: 31.9, 104.5, 105.7, 110.2, 112.1, 116.9, 118.4, 129.5, 133.5, 137.8, 142.4, 143.8, 145.0, 148.2, 149.6, 156.9. Anal. Calculated for C_16_H_11_N_5_OS (MW: 321.36): C, 59.80%; H, 3.45%; N, 21.79%. Found: C, 59.95%; H, 3.58%; N, 21.93%.

##### 3-[6-(3-Methoxy-phenyl)-imidazo[2,1-*b*][1,3,4]thiadiazol-2-yl]-1-methyl-1*H*-pyrrolo[2,3-*b*]pyridine **9m**

Yield: 58%; off-white powder; m.p.: 249 °C; ^1^H NMR (300 MHz, DMSO-*d*_6_) δ: 3.81 (3H, s, CH_3_), 3.90 (3H, s, CH_3_), 6.83–6.91 (1H, m, Ar), 7.27–7.50 (4H, m, Ar), 8.42–8.50 (2H, m, Ar), 8.57 (1H, s, Ar), 8.74 (1H, s, Ar). ^13^C NMR (50 MHz, DMSO-*d*_6_) δ: 32.0, 55.6, 104.4, 110.4, 111.5, 113.6, 116.8, 117.0, 117.5, 118.5, 129.6, 130.3, 133.7, 143.3, 144.4, 144.8, 148.0, 157.2, 160.1. Anal. Calculated for C_19_H_15_N_5_OS (MW: 361.42): C, 63.14%; H, 4.18%; N, 19.38%. Found: C, 63.33%; H, 4.42%; N, 19.59%.

##### 1-Methyl-3-[6-(1,3-thiazol-2-yl)-imidazo[2,1-*b*][1,3,4]thiadiazol-2-yl]-1*H*-pyrrolo[2,3-*b*]pyridine **9n**

Yield: 44%; whitish powder; m.p.: 266 °C. ^1^H NMR (300 MHz, DMSO-*d*_6_) δ: 3.93 (3H, s, CH_3_), 7.23–7.53 (1H, m, Ar), 7.72 (1H, s, Ar), 7.89 (1H, s, Ar), 8.41–8.76 (4H, m, Ar). Anal. Calculated for C_15_H_10_N_6_S_2_ (MW: 338.41): C, 53.24%; H, 2.98%; N, 24.83%. Found: C, 53.41%; H, 2.84%; N, 24.98%.

##### 5-Bromo-3-(6-phenylimidazo[2,1-*b*][1,3,4]thiadiazol-2-yl)-1*H*-pyrrolo[2,3-*b*]pyridine **9o**

Yield: 53%; off-white powder; m.p.: 278 °C. ^1^H NMR (300 MHz, DMSO-*d*_6_) δ: 7.20–7.52 (3H, m, Ar), 7.87 (2H, t, *J* = 5.2 Hz, Ar), 8.38–8.68 (3H, m, Ar), 8.74 (1H, s, Ar), 12.86 (1H, s, NH). ^13^C NMR (50 MHz, DMSO-*d*_6_) δ: 105.5, 111.1, 113.4, 118.3, 125.0 (x2), 127.7, 129.2 (x2), 131.1, 131.9, 134.2, 143.6, 145.3, 145.3, 147.7, 156.7. Anal. Calculated for C_17_H_10_ BrN_5_S (MW: 396.27): C, 51.53%; H, 2.54%; N, 17.67%. Found: C, 51.80%; H, 2.69%; N, 17.83%.

##### 5-Bromo-3-[6-(4-fluoro-phenyl)-imidazo[2,1-*b*][1,3,4]thiadiazol-2-yl]-1*H*-pyrrolo[2,3-b]pyridine **9p**

Yield: 43%; whitish powder; m.p.: 281 °C. ^1^H NMR (300 MHz, DMSO-*d*_6_) δ: 7.33–7.20 (2H, m, Ar), 7.97–7.83 (2H, m, Ar), 8.54–8.46 (1H, m, Ar), 8.60 (2H, dd, *J* = 11.9, 2.6 Hz, Ar), 8.75 (1H, s, Ar), 12.90 (1H, s, NH). ^13^C NMR (50 MHz, DMSO-*d*_6_) δ: 105.4, 110.9, 113.4, 114.5, 115.9, 118.3, 120.6, 124.3, 125.7, 126.5, 126.8, 136.3, 139.9, 147.7, 154.9, 161.3, 165.5. Anal. Calculated for C_17_H_19_BrFN_5_S (MW: 414.26): C, 49.29%; H, 2.19%; N, 16.91%. Found: C, 49.42%; H, 2.36%; N, 17.22%.

##### 5-Bromo-3-[6-(2,5-dimethoxy-phenyl)-imidazo [2,1-*b*][1,3,4]thiadiazol-2-yl]-1*H*-pyrrolo [2,3-*b*]pyridine **9q**

Yield: 51%; off-white powder; m.p.: 300 °C. ^1^H NMR (300 MHz, DMSO-*d*_6_) δ: 3.77 (3H, s, CH_3_), 3.93 (3H, s, CH_3_), 6.85 (1H, dd, *J* = 8.9, 3.2 Hz, Ar), 7.04 (1H, d, *J* = 9.0 Hz, Ar), 7.71 (1H, d, *J* = 3.2 Hz, Ar), 8.49 (1H, d, *J* = 2.3 Hz, Ar), 8.58 (2H, d, *J* = 2.3 Hz, Ar), 8.66 (1H, s, Ar), 12.88 (1H, s, NH). ^13^C NMR (50 MHz, DMSO-*d*_6_) δ: 55.8, 56.2, 99.9, 105.5, 112.1,. 113.0, 113.4, 114.5, 118.4, 123.0, 131.2, 131.8, 140.8, 142.7, 145.3, 147.7, 150.4, 153.7, 156.5. Anal. Calculated for C_19_H_14_BrN_5_O_2_S (MW: 456.32): C, 50.01%; H, 3.09%; N, 15.35%. Found: C, 50.28%; H, 3.25%; N, 15.51%.

##### 5-Bromo-3-[6-(3-methoxy-phenyl)-imidazo[2,1-*b*][1,3,4]thiadiazol-2-yl]-1*H*-pyrrolo[2,3-*b*]pyridine **9r**

Yield: 44%; whitish powder; m.p.: 305 °C; ^1^H NMR (300 MHz, DMSO-*d*_6_) δ: 3.87 (3H, s, CH_3_), 6.88 (1H, s, Ar), 7.41 (3H, d, *J* = 36.3 Hz, Ar), 8.38–8.92 (4H, m, Ar), 12.88 (1H, s, NH). ^13^C NMR (50 MHz, DMSO-*d*_6_) δ: 55.5, 105.5, 110.3 (x2), 111.4, 113.3, 113.3, 117.4 (x2), 118.3, 130.2, 131.1, 131.8, 135.7, 145.3, 145.4, 147.7, 160.1. Anal. Calculated for C_18_H_12_BrN_5_OS (MW: 426.29): C, 50.72%; H, 2.84%; N, 16.43%. Found: C, 50.92%; H, 2.98%; N, 16.70%.

##### 5-Bromo-3-[6-(2,3-dihydro-benzo[1,4]dioxin-6-yl)-imidazo[2,1-*b*][1,3,4]thiadiazol-2-yl]-1*H*-pyrrolo[2,3-*b*]pyridine **9s**

Yield: 45%; off-white powder; m.p.: 304 °C. ^1^H NMR (300 MHz, DMSO-*d*_6_): 4.17–4.41 (4H, m, CH_2_), 6.09 (2H, s, Ar), 7.11 (1H, d, *J* = 8.4 Hz), 7.55–7.66 (2H, m, Ar), 8.43–8.54 (1H, m, Ar), 8.65 (d, *J* = 3.0 Hz, 1H), 12.94 (1H, s, NH). ^13^C NMR (50 MHz, DMSO-*d*_6_): δ: 64.4, 65.6, 103.7, 114.1, 118.0, 123.4, 127.9, 131.2, 132.9, 143.8, 145.6, 147.3, 149.7, 166.7. Anal. Calculated for C_19_H_12_BrN_5_O_2_S (MW: 454.30): C, 50.23%; H,2.66%; N, 15.42%. Found: C, 50.51%; H,2.90%; N, 15.58%.

##### 5-Bromo-3-[6-(furan-2-yl)imidazo[2,1-*b*][1,3,4]thiadiazol-2-yl]-1*H*-pyrrolo[2,3-*b*]pyridine **9t**

Yield: 55%; off-white powder; m.p.: 325 °C. ^1^H NMR (300 MHz, DMSO-*d*_6_) δ: 6.66 (2H, d, *J* = 3.2 Hz, Ar), 7.72 (1H, s, Ar), 8.55 (4H, d, *J* = 35.8 Hz, Ar), 12.92 (1H, s, NH). ^13^C NMR (50 MHz, DMSO-*d*_6_) δ: 105.01, 105.79, 110.7, 112.08, 113.38, 126.81, 131.14, 131.89, 142.48, 143.10, 144.20, 145.36, 146.83, 147.80, 148.50. Anal. Calculated for C_15_H_8_BrN_5_OS (MW: 386.23): C, 46.65%; H, 2.09%; N, 18.13%. Found: C, 46.86%; H, 2.23%; N, 18.31%.

##### 5-Bromo-3-[6-(thiophen-3-yl)imidazo[2,1-*b*][1,3,4]thiadiazol-2-yl]-1*H*-pyrrolo[2,3-*b*]pyridine **9u**

Yield: 48%; whitish powder; m.p.: 298 °C. ^1^H NMR (300 MHz, DMSO-*d*_6_) δ: 7.51 (1H, dd, *J* = 5.0, 1.3 Hz, Ar), 7.71–7.60 (2H, m, Ar), 7.94 (1H, dd, *J* = 2.7, 1.6 Hz, Ar), 8.47 (1H, d, *J* = 2.3 Hz, Ar), 8.65–8.53 (2H, m, Ar), 12.91 (1H, s, NH). ^13^C NMR (50 MHz, DMSO-*d*_6_): 105.3, 113.4, 118.3, 120.2, 121.9, 126.3, 127.4, 131.1, 132.2, 134.6, 138.9, 142.6, 145.4, 147.7, 157.2. Anal. Calculated for C_15_H_8_BrN_5_S_2_ (MW: 402.29): C, 44.79%; H, 2.00%; N, 17.41%. Found: C, 44.91%; H, 2.23%; N, 17.58%.

### 3.2. Biology

#### 3.2.1. Determination of Minimum Inhibitory Concentrations (MICs)

Minimum inhibitory concentrations (MICs) were determined using the broth microdilution method in 96-well microtiter plates, following standardized protocols [32]. Serial two-fold dilutions of each compound were prepared in Mueller–Hinton broth, starting from a stock solution of 5 mg/mL in dimethyl sulfoxide (DMSO), yielding final concentrations ranging from 200 to 0.7 μg/mL. The antimicrobial activity was evaluated against the reference bacterial strains *Staphylococcus aureus* ATCC 25923, *Pseudomonas aeruginosa* ATCC 15442 [33].

Bacterial suspensions were prepared in 0.9% NaCl and adjusted to 1 × 10^6^ CFU/mL by serial dilution of cultures previously grown for 24 h at 37 °C on Tryptic Soy Agar (TSA). Each well containing the test compound received 10 μL of bacterial suspension. Control wells included: a positive control (bacterial suspension without test compound), a negative control (medium only, without inoculum), a DMSO control (medium and DMSO at 2% *v*/*v* or less in according to tested concentrations of samples, to exclude biological activity by diluent) and a substance control (compound in broth without bacterial cells) to account for intrinsic absorbance. The experiments were performed at least in triplicate, and three independent experiments were run. Plates were incubated at 37 °C for 24 h, and bacterial growth was assessed by measuring the optical density (OD) at 570 nm using a microplate reader (GloMax^®^ Multi Detection System, Promega, Milan, Italy). The MIC was defined as the lowest concentration of the compound that resulted in an OD value comparable to that of the negative control.

#### 3.2.2. Anti-Biofilm Activity

The bacterial suspensions of *S. aureus* and *P. aeruginosa* were prepared, inoculating one loopful from a culture, grown at 37 °C for 24 h on TSA, into 5 mL of Tryptic Soy Broth (TSB) supplemented with 2% (*w*/*v*) glucose. The bacterial cultures were incubated at 37 °C for 24 h to promote biofilm formation [34]. Following incubation, 2.5 μL of each bacterial suspension, containing ~1 × 10^6^ CFU/mL, was inoculated into flat-bottom 96-well polystyrene microplates containing 200 μL of TSB with 2% glucose. Test compounds were added to the wells at concentrations ranging from 12.5 to 100 μg/mL. A positive control to check microbial growth (consisting of tested microorganisms in the medium with 2% glucose, without compounds) and a negative control to check the medium sterility (represented by the medium without bacterial suspension) were also included in the 96-well plate. In addition, compound control was also included, consisting of culture medium and the tested compounds in the absence of bacterial inoculum. Amikacin was included as a positive control in the inhibition of biofilm formation assay and evaluated at sub-MICs ranging from 0.5 to 0.01 μg/mL for *P. aeruginosa* and from 2 to 0.06 μg/mL for *S. aureus*.

Post-incubation, non-adherent cells were removed by washing each well twice with sterile 0.9% NaCl solution. The remaining sessile biomass was fixed and stained with 100 μL of 0.1% (*w*/*v*) crystal violet for 30 min at 37 °C. Excess stain was discarded, and wells were gently rinsed twice with tap water. Bound dye was then solubilized by adding 200 μL of ethanol to each well and incubating for 10 min at room temperature. Biofilm quantification was performed by measuring the optical density at 540 nm using a microplate reader (GloMax^®^ Multi Detection System TM297, Promega, Milan, Italy).

All experiments were conducted in triplicate and independently repeated at least three times. The percentage of biofilm inhibition was calculated according to the following formula:

% of inhibition = (OD growth control − OD sample)/OD growth control) × 100

The BIC_50_ value, defined as the concentration of the compound required to inhibit biofilm formation by 50%, was determined for each treatment condition and the value was calculated using AAT Quest GraphTM IC50 Calculator (v.1) (Bioquest, Inc., Sunnyvale, CA, USA) retrieved from https://www.aatbio.com/tools/ic50-calculator (accessed on 16 February 2026). Data are presented as mean values ± standard deviations (SD) derived from three independent experiments, each performed in triplicate. Statistical significance between treated and control groups was assessed using Student’s *t*-test, with a significance threshold set at *p* < 0.005.

#### 3.2.3. Scanning Electron Microscopy (SEM)

The bacterial suspension of *S. aureus* was prepared, inoculating one loopful from a culture, grown at 37 °C for 24 h on TSA, into TSB containing 2% *v*/*v* glucose. The bacterial culture was incubated overnight at 37 °C. After incubation time, 250 μL of bacterial suspension of *S. aureus*, containing ~1 × 10^6^ CFU/mL, was inoculated into flat-bottom 24-well polystyrene microplates containing 2 mL of TSB with 2% glucose. Sterile glass slides were placed into the wells of flat-bottom 24-well polystyrene microplates prior to inoculation. Subsequently, the compounds **9g** and **9u** were added to the wells at the concentration of 100 µg/mL. Growth control wells containing microorganisms without any compounds and a negative control consisting of culture broth without microorganisms were performed for comparison under the same experimental conditions. The microplates were then incubated at 37 °C to promote biofilm formation on the surface of the glass slides. After this time, the medium was discarded and the slides inside the wells were washed three times with 1 mL of sterile NaCl (0.9% *v*/*v* solution) to remove non-adherent cells.

Biofilm formation of *S. aureus* ATCC 25923 was evaluated by scanning electron microscopy (SEM; Phenom ProX, PhenomWorld, Eindhoven, The Netherlands) using the method described by Bezerra et al. Biofilms formed on the glass slides were fixed with a 4% formaldehyde solution in DPBS for 30 min at 37 °C. Subsequently, samples were gently washed three times with sodium phosphate buffer (pH 7.4). The biofilms were then dehydrated through a graded ethanol series (30%, 50%, 70%, and 100%) for 10 min at 24 °C before SEM analysis. SEM observations were performed to assess biofilm development and surface coverage as an indicator of biofilm biomass.

#### 3.2.4. Effect of Compounds Against Preformed Biofilm Biomasses

Preformed biofilm destruction assays were conducted using the reference bacterial strains *S. aureus* ATCC 25923 and *P. aeruginosa* ATCC 15442. The strains were cultured under the conditions previously described. Untreated bacterial cultures and uninoculated medium were used as positive (growth) and negative (sterility) controls, respectively. Amikacin was used as a positive control in the pre-formed biofilm eradication assay and tested at a concentration corresponding to 2× MIC for each bacterial strain (8 μg/mL for *S. aureus* and 2 μg/mL for *P. aeruginosa*.)

Biofilms were established in 96-well flat-bottom microtiter plates as outlined above. After 24 h of incubation at 37 °C, non-adherent planktonic cells were carefully aspirated, and wells were gently washed with 200 μL of sterile 0.9% NaCl solution. Test compounds were then applied at concentrations ranging from 10 to 200 μg/mL, and plates were further incubated for 24 h at 37 °C.

Biofilm biomass quantification was performed by crystal violet staining, as detailed in Section 3.2.2. All results are expressed as mean ± standard deviation (SD) from three independent experiments, each conducted in three technical replicates per experiment (individual wells within the same plate). Statistical analysis was performed using Student’s *t*-test, with differences considered significant at *p* < 0.005.

### 3.3. In Vivo Anti-Infective Activity and Toxicity Evaluation of Compounds ***9g*** and ***9u***

#### 3.3.1. Insect Rearing and Preparation

Larvae of the greater wax moth *Galleria mellonella* L. (Lepidoptera: Pyralidae) were reared on a natural diet consisting of honeybee nest debris and maintained at 30 °C under dark conditions. Final instar larvae displaying a creamy coloration, with a mean body weight of 401 ± 10 mg and an average length of approximately 2.5 cm, were selected for the experiments. Prior to the assays, larval health and quality were carefully assessed. Before the *in vivo* bioassay, larvae were washed with distilled water and surface-sterilized by brief immersion in 70% (*v*/*v*) ethanol.

#### 3.3.2. *In Vivo* Bioassay

The protective activity of compounds **9g** and **9u** against bacterial infection was evaluated in *G. mellonella* larvae using an established *in vivo* model. *S. aureus* ATCC 29213 was used as the pathogenic strain. Each larva was injected with 10 µL of a bacterial suspension corresponding to 2.8 × 10^4^ CFU/mL. Simultaneously, larvae received a single treatment dose of the tested compound (1 mg/kg), dissolved in 0.9% (*w*/*v*) NaCl solution.

Two uninfected control groups were included: an untreated group and a group injected with 0.9% (*w*/*v*) NaCl containing DMSO used to dissolve the tested compounds. Two infected positive control groups were established by inoculating larvae with *S. aureus* (2.8 × 10^4^ CFU/mL) in the absence of antibacterial agents. Two additional groups of infected larvae received a single dose (1 mg/kg) of compounds **9g** or **9u**.

Each experimental group consisted of six replicates. In total, 42 larvae were used, including one group that received no treatment. Larvae were inoculated by injection between the third and fourth left prolegs using a 100 µL Hamilton syringe. To minimize stress caused by repeated punctures on the same side of the body, the first injection was performed on the left side and the second on the right side. Following inoculation, larvae were incubated at 37 °C in the dark and monitored for survival and melanization (dark pigmentation) at 6, 24, 48, 72, and 82 h for up to 96 h, unless otherwise specified.

#### 3.3.3. Insect Data Analysis

All statistical analyses were performed using Statistica 6.0 (StatSoft, Tulsa, OK, USA). A significance level of α = 0.05 was applied. Larval mortality was corrected using Abbott’s formula. Differences among experimental groups were evaluated by one-way analysis of variance (ANOVA), followed by Tukey’s post hoc test for comparisons between treatment groups and the corresponding controls. In addition, Kaplan–Meier survival analysis was performed, and survival curves were compared using the Mantel–Cox log-rank test.

## 4. Conclusions

Biofilms are widely recognized as key virulence factors responsible for serious bacterial chronic infections that are very difficult to treat with conventional antibiotics. The control of infectious diseases associated with the formation of biofilms remains a challenge that must be met and addressed effectively and urgently.

Here we report the synthesis and the anti-biofilm activity of a new class of twenty-one imidazo[2,1-*b*][1,3,4]thiadiazol-2-yl)-1*H*-pyrrolo[2,3-*b*]pyridines. The new compounds displayed a typical anti-virulence behavior, hindering the sessile form of life without affecting bacterial viability. Through this mechanism of action, it is possible to disarm pathogens without triggering the development of dangerous resistance mechanisms. The new compounds proved to be potent inhibitors of *S. aureus* biofilm, in particular derivatives **9g**, **9i**, **9n**, **9q** and **9u**, which showed BIC_50_ values lower than 15 µg/mL.

*In vivo* studies identified compound **9u** as the most promising derivative, showing lower toxicity and greater protection against staphylococcal infection in the *G. mellonella* model, especially during the early stages of infection. Considering the high pathogenicity of the bacterial strain used, the protective effect observed within the first 24 h is noteworthy.

Further studies are needed to better define the pharmacological profile of compound **9u**, including evaluation at lower doses and through alternative routes of administration.

Notably, derivative **9h** was able both to inhibit biofilm formation and to disrupt mature biofilms, making it an interesting lead compound for the development of new anti-biofilm agents. Since *S. aureus* biofilms play a central role in chronic and antibiotic-resistant infections, compounds targeting both biofilm formation and established biofilms may represent valuable therapeutic tools. Moreover, anti-virulence agents used alone or in combination with antibiotics could enhance the susceptibility of pathogens to conventional treatments, offering promising strategies against resistant *S. aureus* infections.

## Figures and Tables

**Table 1:** New 3-(6-phenyl-imidazo[2,1-*b*][1,3,4]thiadiazol-2-yl)-1*H*-pyrrolo[2,3-*b*]pyridines **9a**–**u**.

Compounds	R	R^1^	R^2^
**9a**	H	H	3-OCH_3_-Ph
**9b**	H	H	4F-Ph
**9c**	H	H	3-thiophenyl
**9d**	H	H	3-pyridinyl
**9e**	H	H	2-thiazolyl
**9f**	H	CH_3_	Ph
**9g**	H	CH_3_	2,5-OCH_3_-Ph
**9h**	H	CH_3_	3-thiophenyl
**9i**	H	CH_3_	3-pyridinyl
**9j**	H	CH_3_	4F-Ph
**9k**	H	CH_3_	2,3-dihydro-benzo[1,4]dioxin-6-yl
**9l**	H	CH_3_	2-furanyl
**9m**	H	CH_3_	3-OCH_3_-Ph
**9n**	H	CH_3_	2-thiazolyl
**9o**	Br	H	Ph
**9p**	Br	H	4F-Ph
**9q**	Br	H	2,5-OCH_3_-Ph
**9r**	Br	H	3-OCH_3_-Ph
**9s**	Br	H	2,3-dihydro-benzo[1,4]dioxin-6-yl
**9t**	Br	H	2-furanyl
**9u**	Br	H	3-thiophenyl

**Table 2:** Mean values and standard deviation (SD) of Inhibition of biofilm formation, expressed as percentage of inhibition (% at 100, 50, 25, 12.5 and 6.2 μg/mL for *S. aureus*; % at 100 and 50 μg/mL for *P. aeruginosa*).

Cpd	*S. aureus* ATCC 25923					*P. aeruginosa* ATCC 15442	
	100 μg/mL	50 μg/mL	25 μg/mL	12.5 μg/mL	6.2 μg/mL	100 μg/mL	50 μg/mL
**9a**	43 (±3.2)	22 (±2.1)	20 (±1.7)	12 (±1.2)	n.s.	n.s.	n.s.
**9b**	77 (±4.5)	51 (±3.2)	46 (±2.8)	42 (±2.3)	n.s.	16 (±1.5)	n.s.
**9c**	66 (±3.7)	19 (±1.9)	18 (±1.4)	n.s.	n.s.	n.s.	n.s.
**9d**	67 (±4.1)	56 (±3.5)	51 (±3.1)	48 (±2.7)	n.s.	32 (±3.0)	n.s.
**9e**	69 (±3.6)	50 (±2.7)	39 (±2.2)	13 (±1.5)	n.s.	20 (±1.8)	n.s.
**9f**	71 (±4.2)	54 (±3.0)	51 (±3.2)	43 (±2.4)	n.s.	n.s.	n.s.
**9g**	73 (±4.8)	66 (±3.7)	57 (±3.3)	54 (±2.9)	46 (±2.5)	27 (±2.4)	17 (±1.9)
**9h**	73 (±3.9)	43 (±2.2)	42 (±2.1)	32 (±2.0)	n.s.	n.s.	n.s.
**9i**	74 (±4.0)	55 (±3.0)	52 (±2.8)	45 (±2.4)	n.s.	12 (±1.1)	n.s.
**9j**	78 (±4.6)	66 (±3.4)	41 (±2.2)	24 (±1.8)	n.s.	21 (±2.0)	17 (±1.7)
**9k**	64 (±3.3)	50 (±2.3)	13 (±1.2)	9 (±1.0)	n.s.	21 (±2.1)	n.s.
**9l**	77 (±4.4)	64 (±3.5)	50 (±3.1)	34 (±2.5)	n.s.	22 (±2.2)	n.s.
**9m**	62 (±3.1)	38 (±2.0)	37 (±1.8)	n.s.	n.s.	n.s.	n.s.
**9n**	77 (±4.3)	59 (±3.1)	53 (±2.9)	50 (±2.6)	n.s.	26 (±2.5)	20 (±2.0)
**9o**	77 (±4.5)	56 (±3.0)	51 (±2.8)	48 (±2.7)	n.s.	26 (±2.3)	14 (±1.5)
**9p**	63 (±3.2)	56 (±3.0)	34 (±2.0)	41 (±2.1)	n.s.	n.s.	n.s.
**9q**	77 (±4.4)	58 (±3.2)	51 (±3.0)	48 (±2.6)	n.s.	n.s.	n.s.
**9r**	66 (±3.1)	44 (±2.1)	32 (±1.9)	16 (±1.4)	n.s.	24 (±2.0)	22 (±1.8)
**9s**	65 (±3.0)	45 (±2.2)	45 (±2.0)	26 (±1.8)	n.s.	39 (±2.8)	n.s.
**9t**	69 (±3.3)	59 (±3.1)	41 (±2.2)	40 (±2.1)	n.s.	33 (±2.4)	n.s.
**9u**	67 (±3.4)	61 (±3.3)	59 (±3.0)	53 (±2.7)	47 (±2.5)	n.s.	n.s.

n.s. = not significant, less than 10% inhibition.

**Table 3:** Inhibition of biofilm formation against *S. aureus* ATCC 25923, expressed as BIC_50_ (μg/mL).

BIC_50_ (µg/mL)	
Compounds	*S. aureus* ATCC 25923
**9b**	51.8
**9c**	71.7
**9d**	16.5
**9e**	50.0
**9f**	25.4
**9g**	9.2
**9h**	67.7
**9i**	13.9
**9j**	30.3
**9k**	50.0
**9l**	25.0
**9m**	72.3
**9n**	12.5
**9o**	15.9
**9p**	66.3
**9q**	13.0
**9r**	71.5
**9s**	72.7
**9t**	36.4
**9u**	8.3

**Table 4:** Mean values and standard deviation (SD) of anti-biofilm activity of compounds **9a**–**u** against 24 h preformed biofilm of *S. aureus* ATCC 25923, expressed as inhibition percentage (%) at the screening concentrations of 200, 100 and 50 μg/mL. Only derivatives showing inhibition values of 20% or higher are reported.

Compounds	*S. aureus* ATCC 25923.		
	200 μg/mL	100 μg/mL	50 μg/mL
**9a**	n.s.	n.s.	n.s.
**9b**	n.s.	n.s.	n.s.
**9c**	n.s.	n.s.	n.s.
**9d**	n.s.	n.s.	n.s.
**9e**	n.s.	n.s.	n.s.
**9f**	n.s.	n.s.	n.s.
**9g**	n.s.	n.s.	n.s.
**9h**	64 (±3.3)	58 ± (2.4)	57 (±3.3)
**9i**	50 (±2.9)	49 (±3.5)	45 (±2.7)
**9j**	40 (±3.1)	24 (±2.6)	20 (±3.2)
**9k**	57 (±2.2)	46 (±2.3)	45 (±3.2)
**9l**	n.s.	n.s.	n.s.
**9m**	n.s.	n.s.	n.s.
**9n**	39 (±1.9)	34 (±3.1)	22 (±2.6)
**9o**	26 (±2.4)	26 (±2.2)	25 (±2.0)
**9p**	n.s.	n.s.	n.s.
**9q**	41 (±3.1)	45 (±2.0)	46 (±3.7)
**9r**	n.s.	n.s.	n.s.
**9s**	n.s.	n.s.	n.s.
**9t**	n.s.	n.s.	n.s.
**9u**	n.s.	n.s.	n.s.

n.s. = not significant, less than 10% inhibition.

**Table 5:** Dispersal activity of the most potent derivatives **9h**, **9i** and **9k** against 24 h preformed biofilm of *S. aureus* ATCC 25923 expressed as BIC _50_ value (μg/mL).

BIC_50_ (µg/mL)	
Compounds	*S. aureus* ATCC 25923
**9h**	39.7
**9i**	200.0
**9k**	141.7

**Table 6:** Risk ratios (RR) of compounds **9g** and **9u** vs. Control (Ctrl) with 95% confidence interval (CI).

Time	RR (9g vs. Ctrl)	CI 95%	RR (9u vs. Ctrl)	CI 95%
0–3 h	5.000	0.299–83.690	0.857	0.020–37.008
3–6 h	1.500	0.037–61.294	0.857	0.020–37.008
6–24 h	0.500	0.026–9.458	1.429	0.260–7.863
24–48 h	8.750	0.615–124.540	3.000	0.157–57.368
48–72 h	n.e.	-	1.250	0.031–50.203
72–82 h	n.e.	-	1.250	0.031–50.203

n.e.: not estimable in an interpretable way.

## Data Availability

The data presented in this study are available on request from the corresponding author.

## Supplementary Materials

The following supporting information can be downloaded at: https://www.mdpi.com/article/10.3390/antibiotics15060598/s1.

## Abbreviations

The following abbreviations are used in this manuscript:

CI	Confidence interval
DMSO	dimethyl sulfoxide
FDR	False Discovery Rate
IC_50_	Half Maximal Inhibitory Concentration
MDR	multidrug-resistant
MIC	minimum inhibitory concentration
OD	Optical density
RR	Risk ratio
SAR	Structure–activity relationship
SD	Standard deviation
TDA	tris [2- (2-methoxyethoxy) ethyl] amine
TSB	Tryptic Soy Broth
