# Supplementary material for: Imidazo[2,1-b][1,3,4]thiadiazol-2-yl]-1H-pyrrolo[2,3-b]pyridines as Inhibitors of Staphylococcus aureus Biofilm Formation
Source: Antibiotics (Basel). 2026 Jun 12;15(6):598. doi: 10.3390/antibiotics15060598 (PMC13296061; doi:10.3390/antibiotics15060598)
Supplement: Supplementary file 1 [file antibiotics-15-00598-s001.zip › antibiotics-4303269-supplementary.pdf]

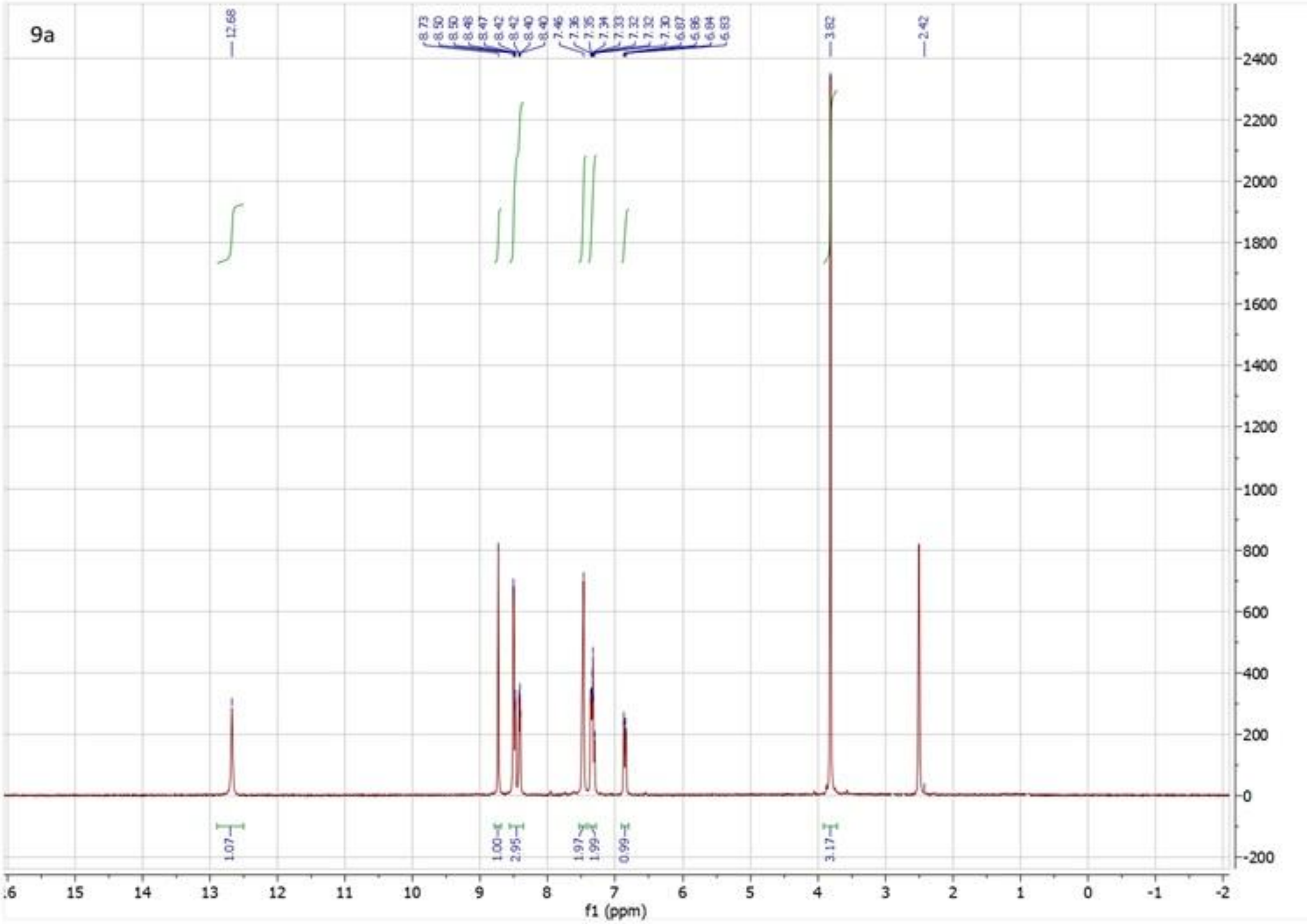

9a
12.68
8.73
8.50
8.50
8.48
8.47
8.42
8.42
8.40
8.40
7.46
7.36
7.35
7.34
7.33
7.32
7.32
7.30
6.87
6.86
6.84
6.83
3.82
2.42
1.07
1.00
2.95
1.97
1.99
0.99
3.17
f1 (ppm)

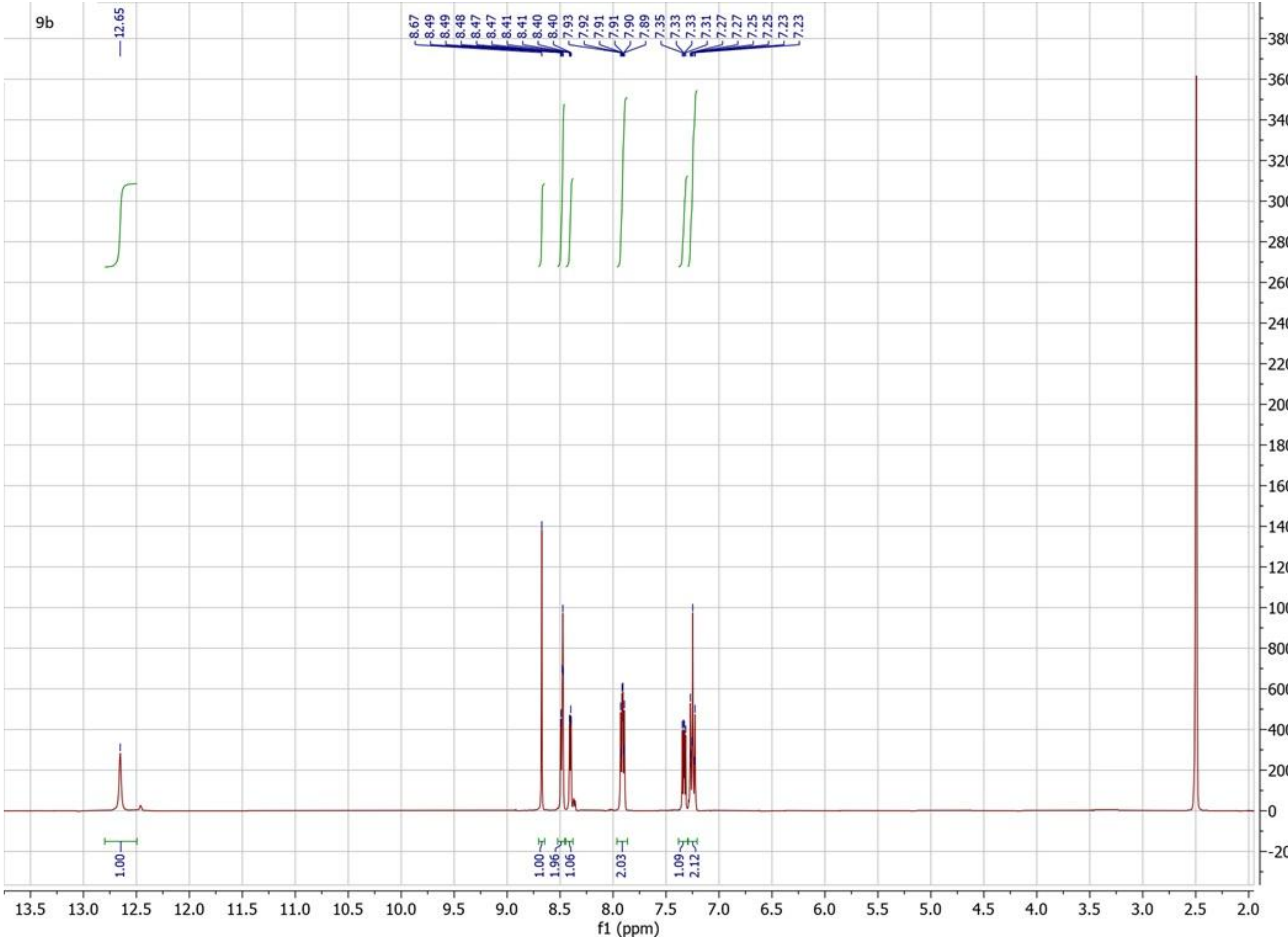
9b
12.65
8.67
8.49
8.49
8.48
8.47
8.47
8.41
8.41
8.40
8.40
7.93
7.92
7.91
7.91
7.90
7.89
7.35
7.33
7.33
7.31
7.27
7.27
7.25
7.25
7.23
7.23
1.00
1.00
1.96
1.06
2.03
1.09
2.12
f1 (ppm)

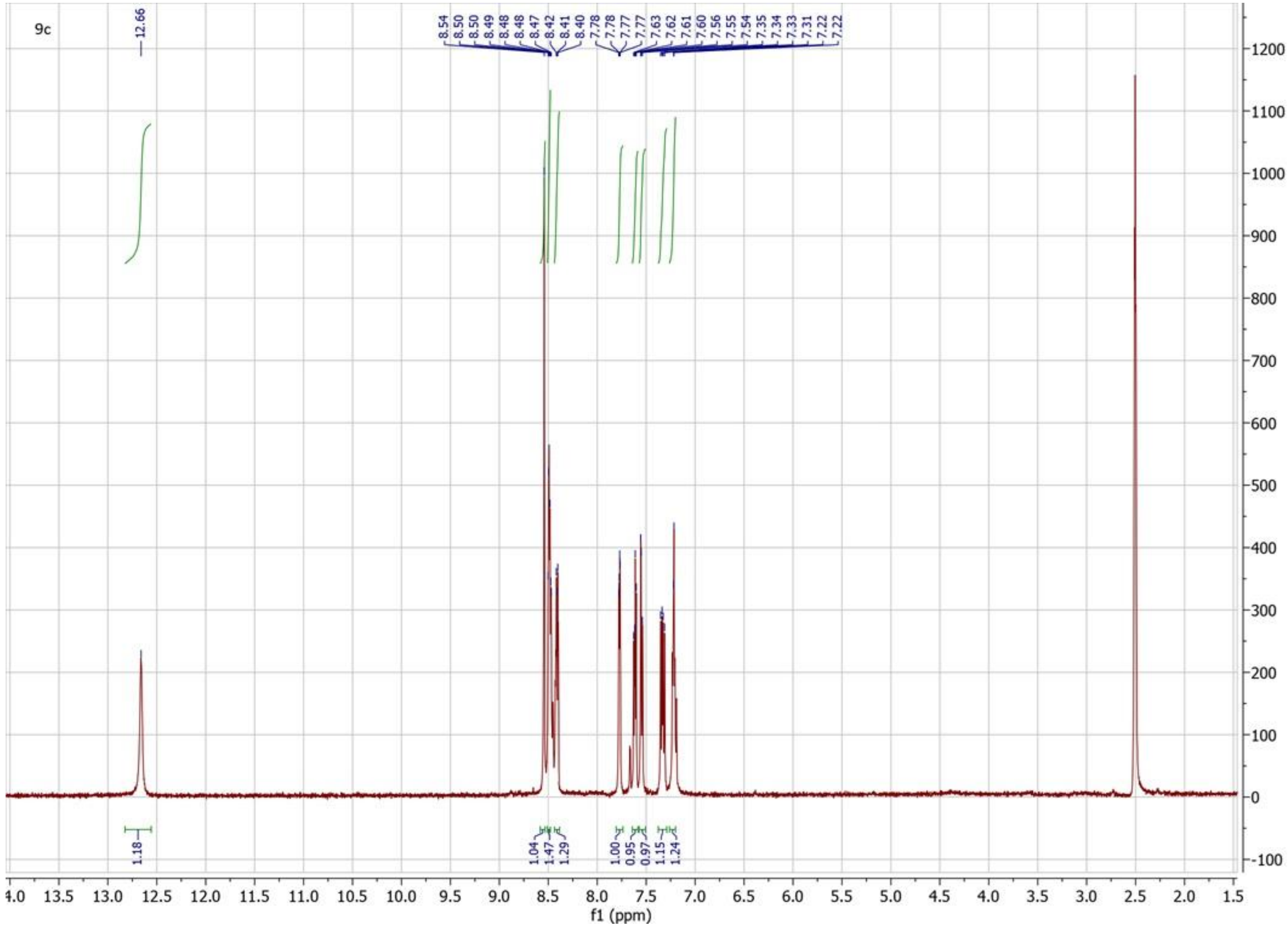
9c
12.66
8.54
8.50
8.50
8.49
8.48
8.48
8.47
8.42
8.41
8.40
7.78
7.78
7.77
7.77
7.63
7.62
7.61
7.60
7.56
7.55
7.54
7.35
7.34
7.33
7.31
7.22
7.22
1.18
1.04
1.47
1.29
1.00
0.95
0.97
1.15
1.24
f1 (ppm)

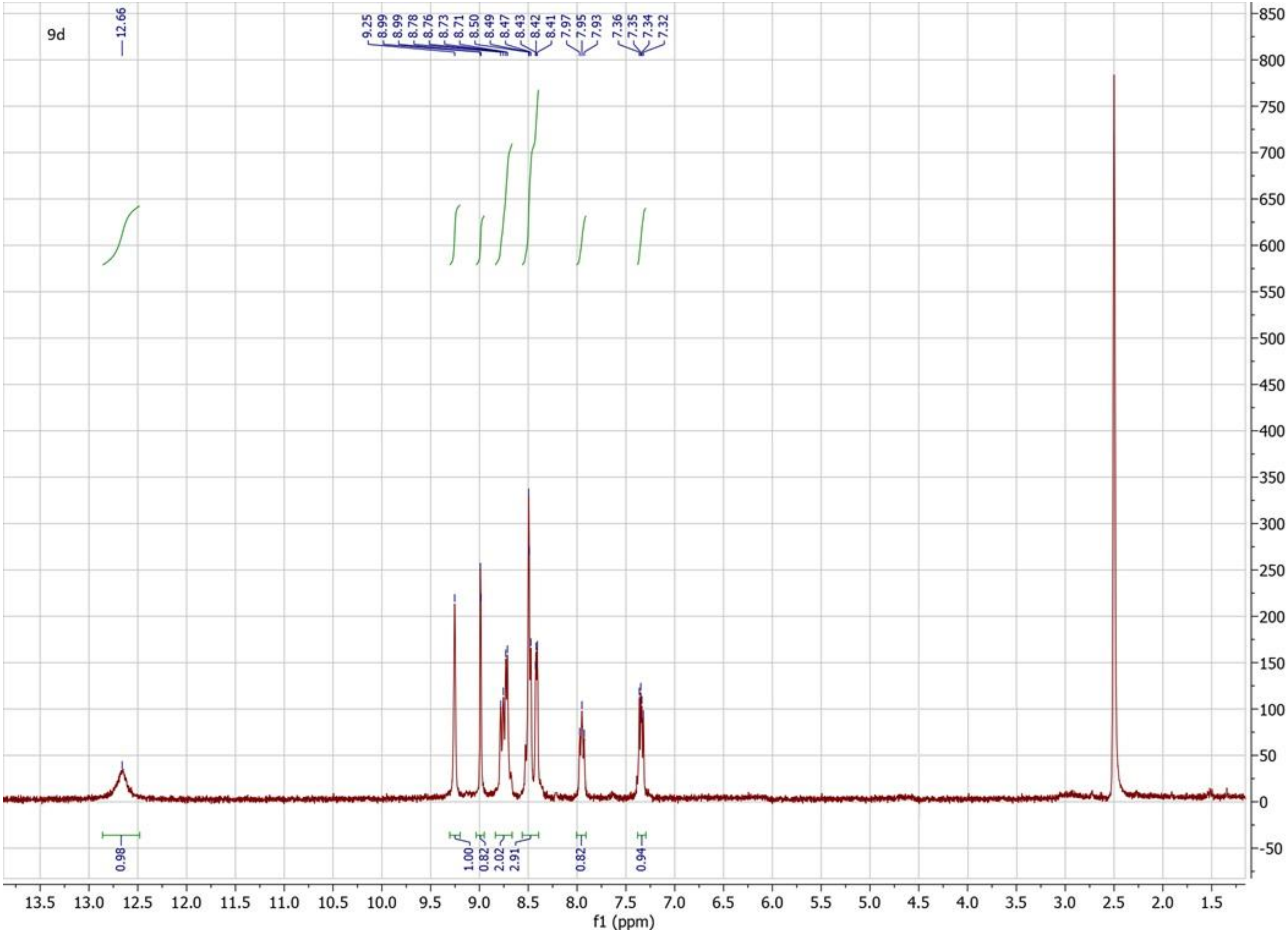
9d
12.66
9.25
8.99
8.99
8.78
8.76
8.73
8.71
8.50
8.49
8.47
8.43
8.42
8.41
7.97
7.95
7.93
7.36
7.35
7.34
7.32
0.98
1.00
0.82
2.02
2.91
0.82
0.94
f1 (ppm)

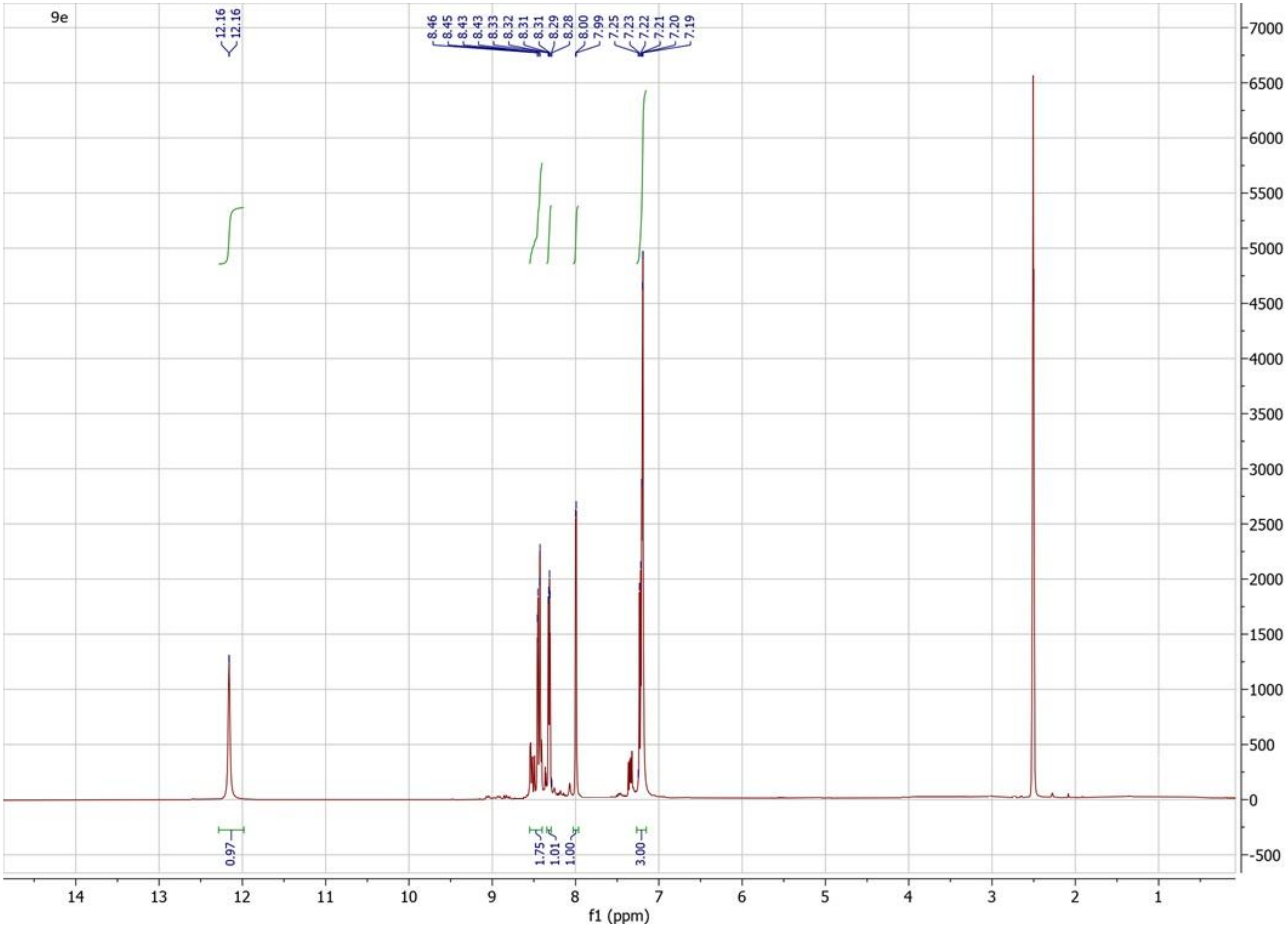

9e
12.16
12.16
8.46
8.45
8.43
8.43
8.33
8.32
8.31
8.31
8.29
8.28
8.00
7.99
7.25
7.23
7.22
7.21
7.20
7.19
0.97
1.75
1.01
1.00
3.00
f1 (ppm)

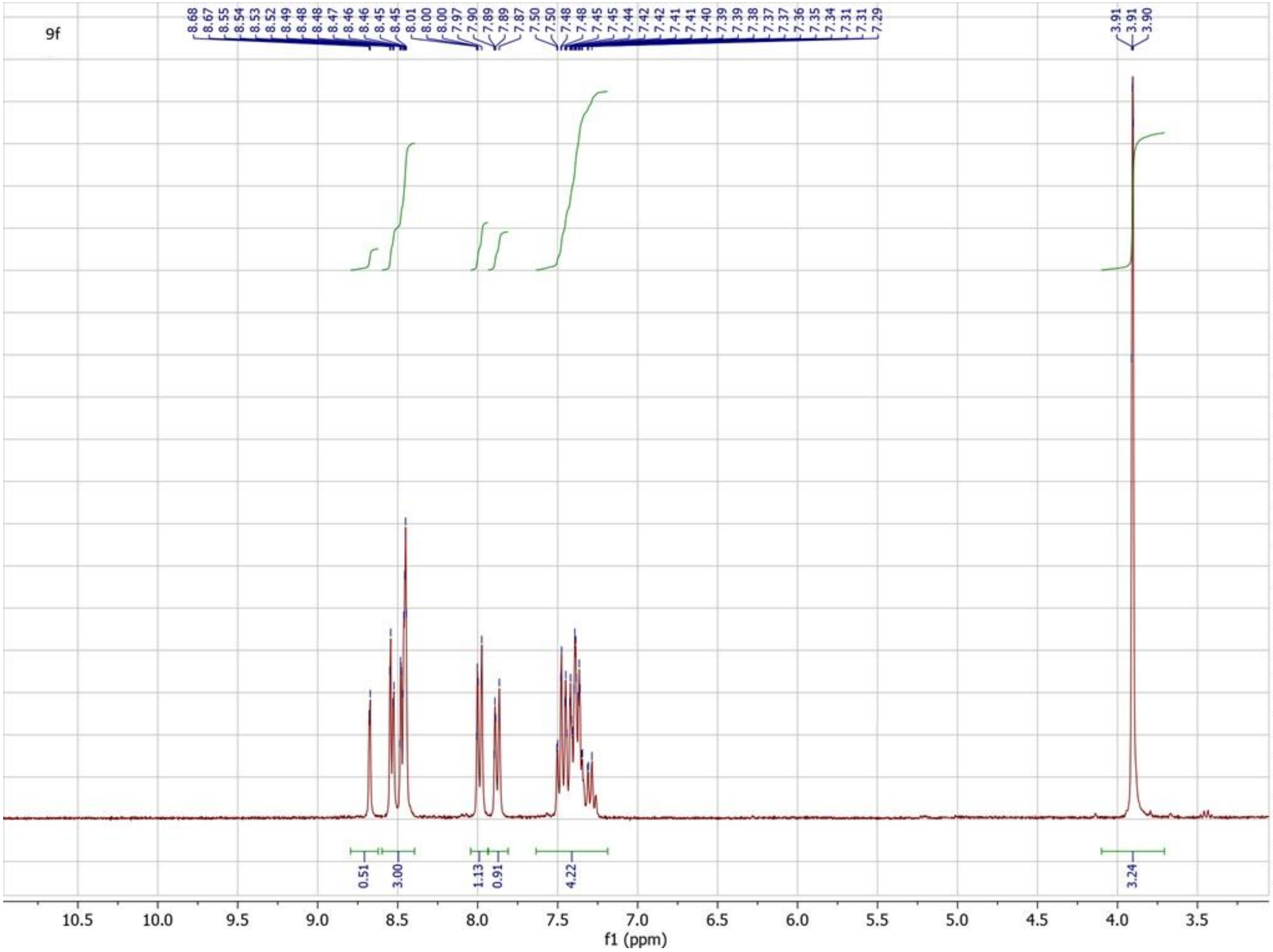

9f
8.68
8.67
8.55
8.54
8.53
8.52
8.49
8.48
8.48
8.47
8.46
8.46
8.45
8.45
8.01
8.00
8.00
7.97
7.90
7.89
7.89
7.87
7.50
7.50
7.48
7.48
7.45
7.45
7.44
7.42
7.42
7.41
7.41
7.40
7.39
7.39
7.38
7.37
7.37
7.36
7.35
7.34
7.31
7.31
7.29
3.91
3.91
3.90
0.51
3.00
1.13
0.91
4.22
3.24
f1 (ppm)

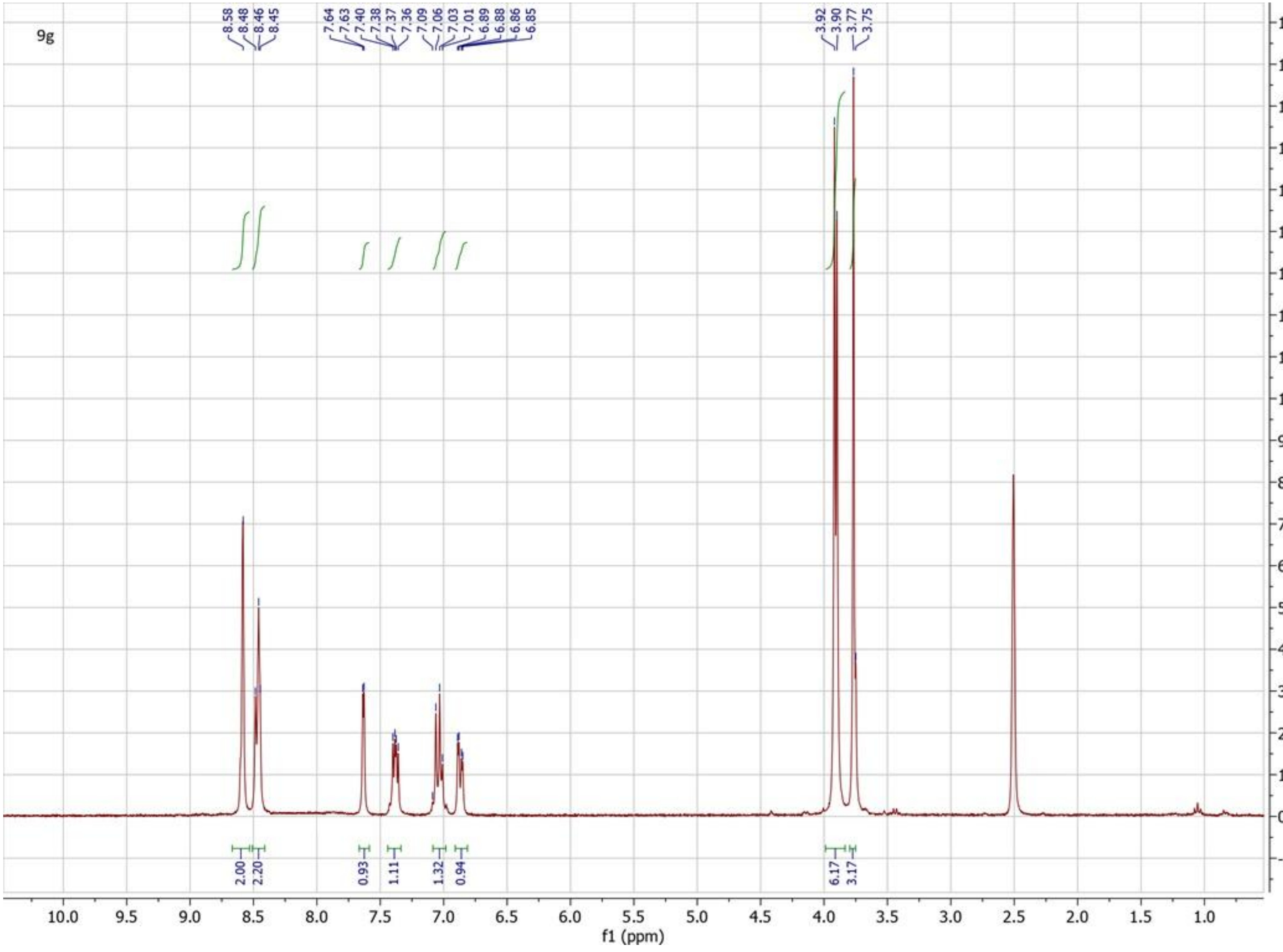
9g
8.58
8.48
8.46
8.45
7.64
7.63
7.40
7.38
7.37
7.36
7.09
7.06
7.03
7.01
6.89
6.88
6.86
6.85
3.92
3.90
3.77
3.75
2.00
2.20
0.93
1.11
1.32
0.94
6.17
3.17
f1 (ppm)

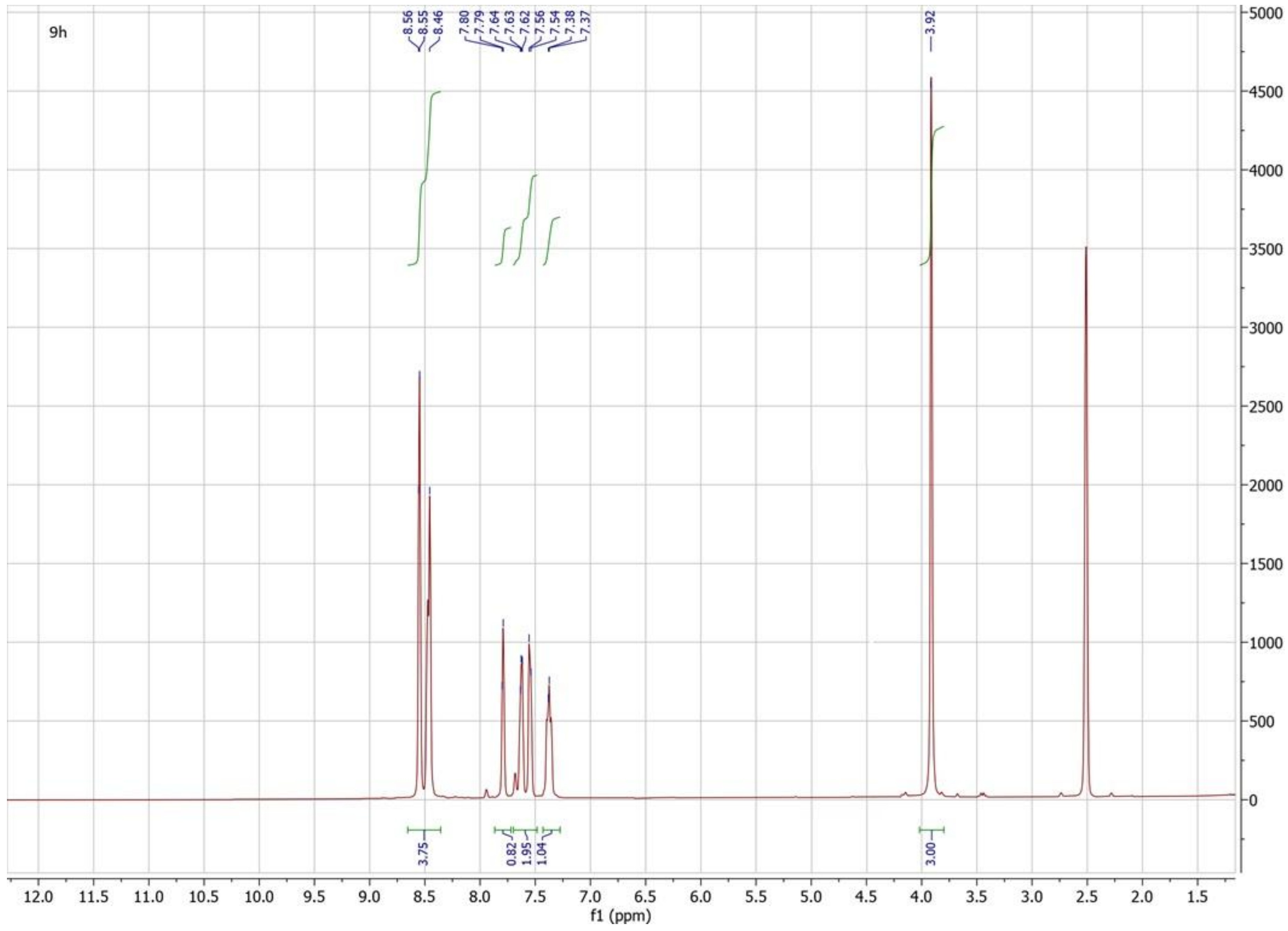
9h
8.56
8.55
8.46
7.80
7.79
7.64
7.63
7.62
7.56
7.54
7.38
7.37
3.92
3.75
0.82
1.95
1.04
3.00
f1 (ppm)

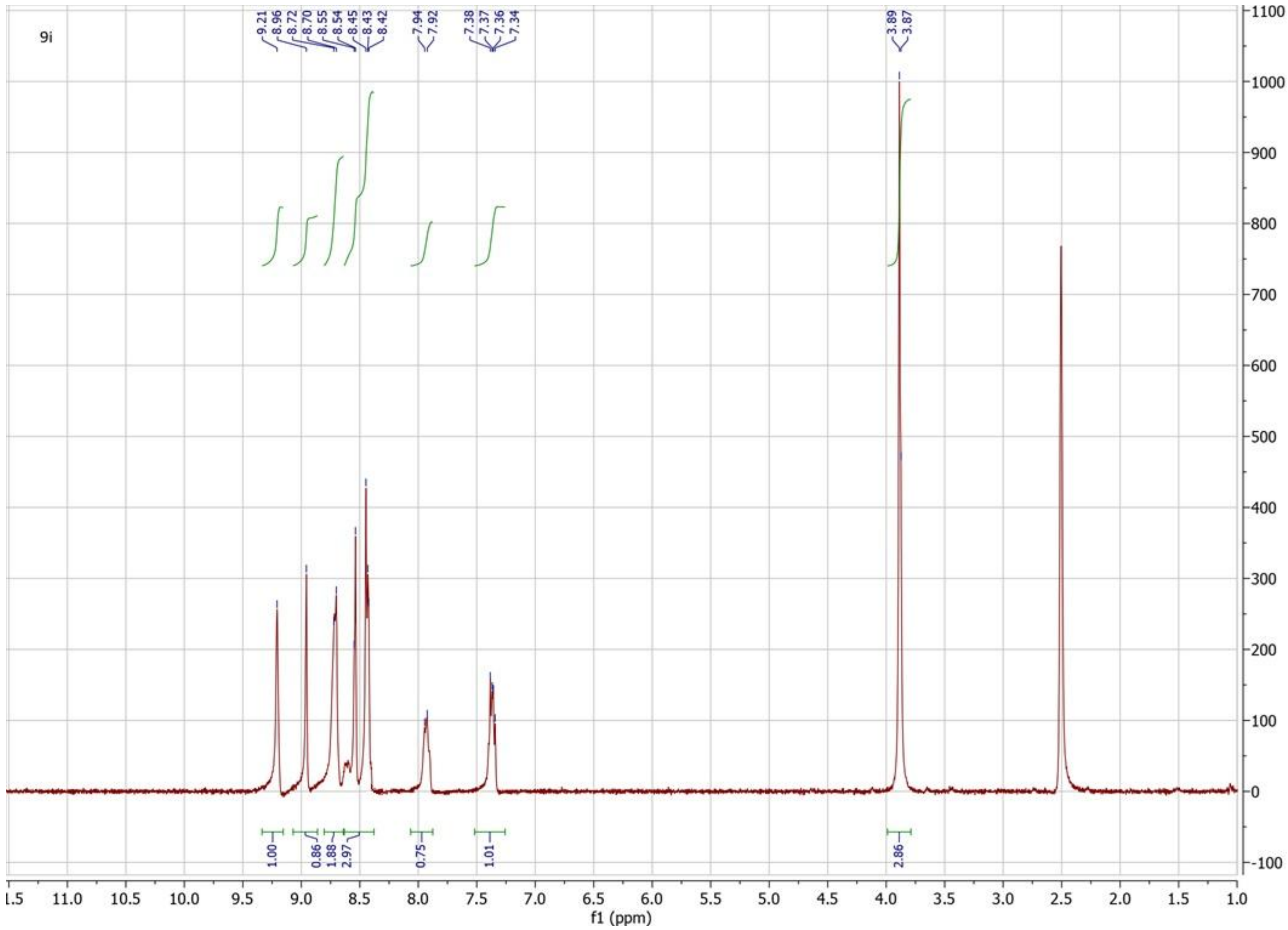

9i
9.21
8.96
8.72
8.70
8.55
8.54
8.45
8.43
8.42
7.94
7.92
7.38
7.37
7.36
7.34
3.89
3.87
1.00
0.86
1.88
2.97
0.75
1.01
2.86
f1 (ppm)

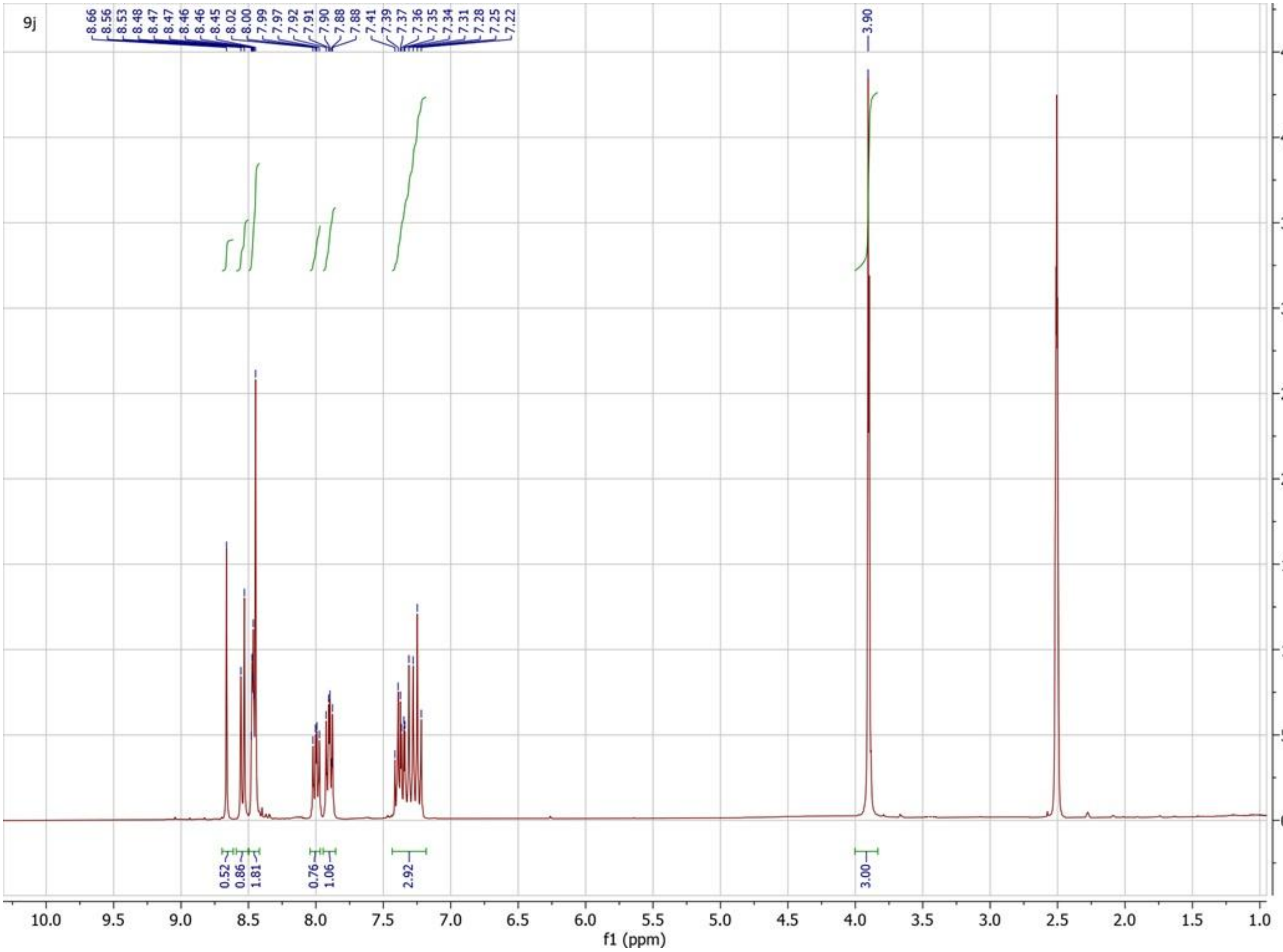

9j
8.66
8.56
8.53
8.48
8.47
8.47
8.46
8.46
8.45
8.02
8.00
7.99
7.97
7.92
7.91
7.90
7.88
7.88
7.41
7.39
7.37
7.36
7.35
7.34
7.31
7.28
7.25
7.22
3.90
0.52
0.86
1.81
0.76
1.06
2.92
3.00
f1 (ppm)

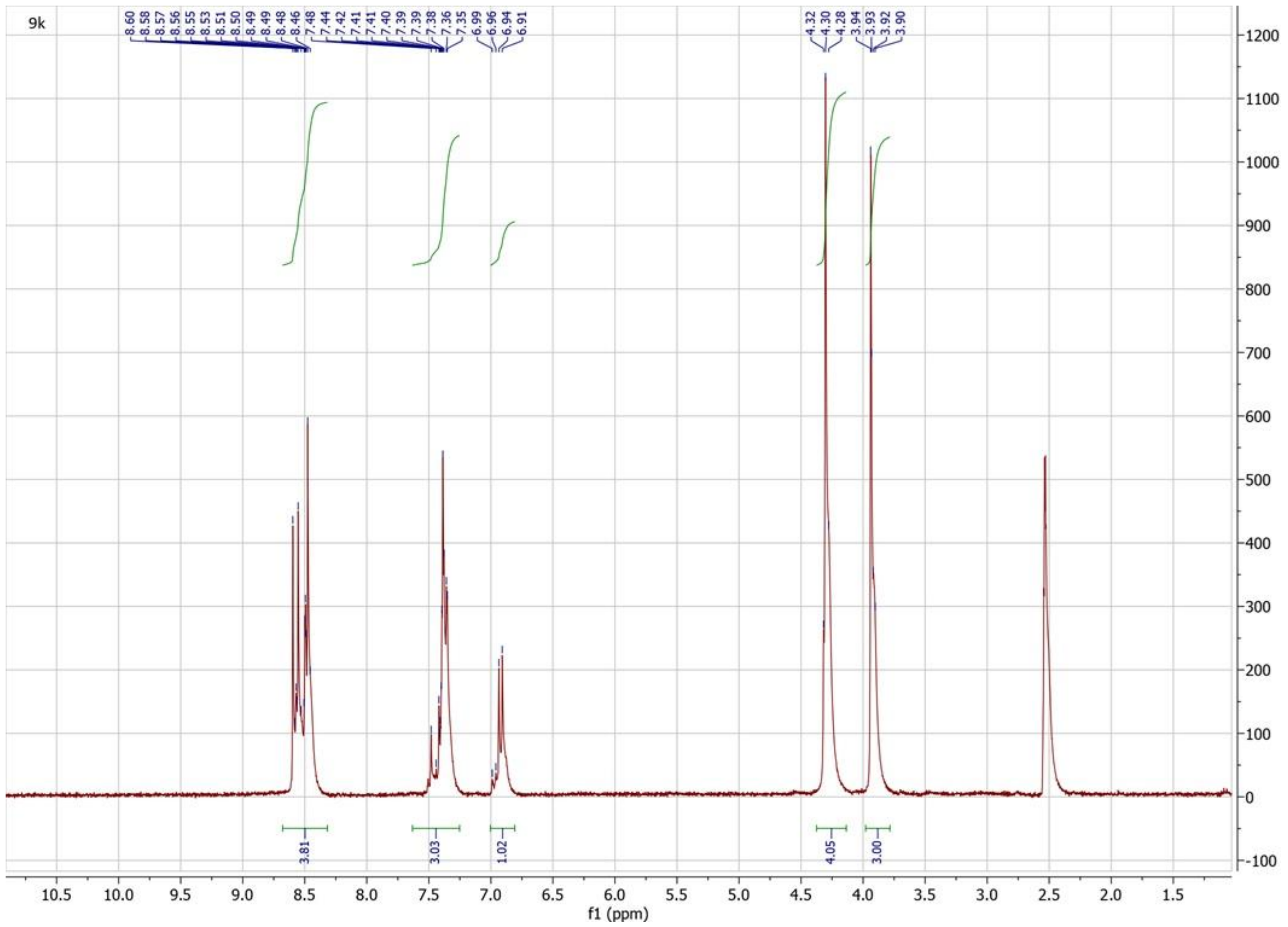
9k
8.60
8.58
8.57
8.56
8.55
8.53
8.51
8.50
8.49
8.49
8.48
8.46
7.48
7.44
7.42
7.41
7.41
7.40
7.39
7.39
7.38
7.36
7.35
6.99
6.96
6.94
6.91
4.32
4.30
4.28
3.94
3.93
3.92
3.90
3.81
3.03
1.02
4.05
3.00
f1 (ppm)

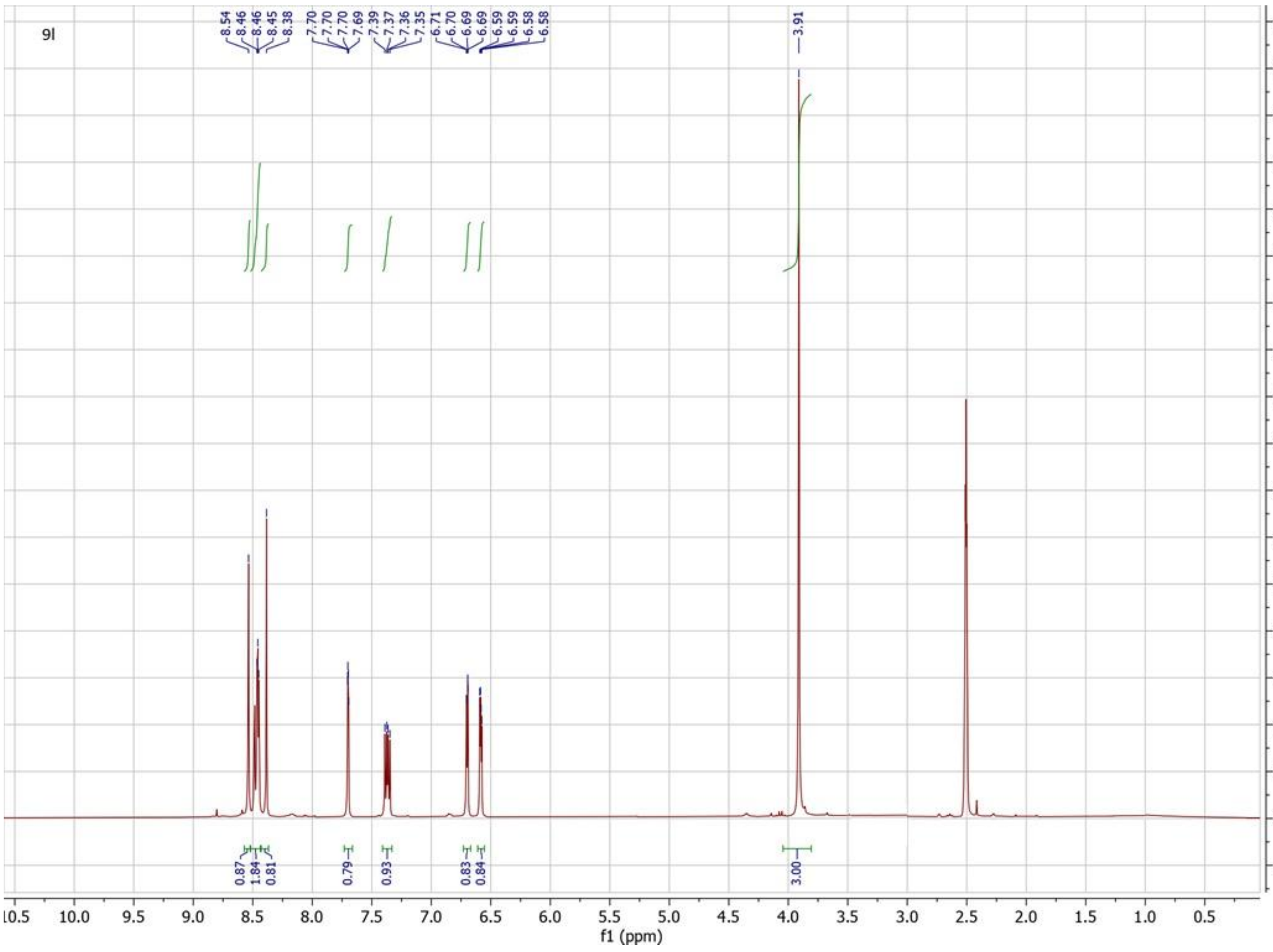
9l
8.54
8.46
8.46
8.45
8.38
7.70
7.70
7.70
7.69
7.39
7.37
7.36
7.35
6.71
6.70
6.69
6.69
6.59
6.59
6.58
6.58
3.91
0.87
1.84
0.81
0.79
0.93
0.83
0.84
3.00
f1 (ppm)

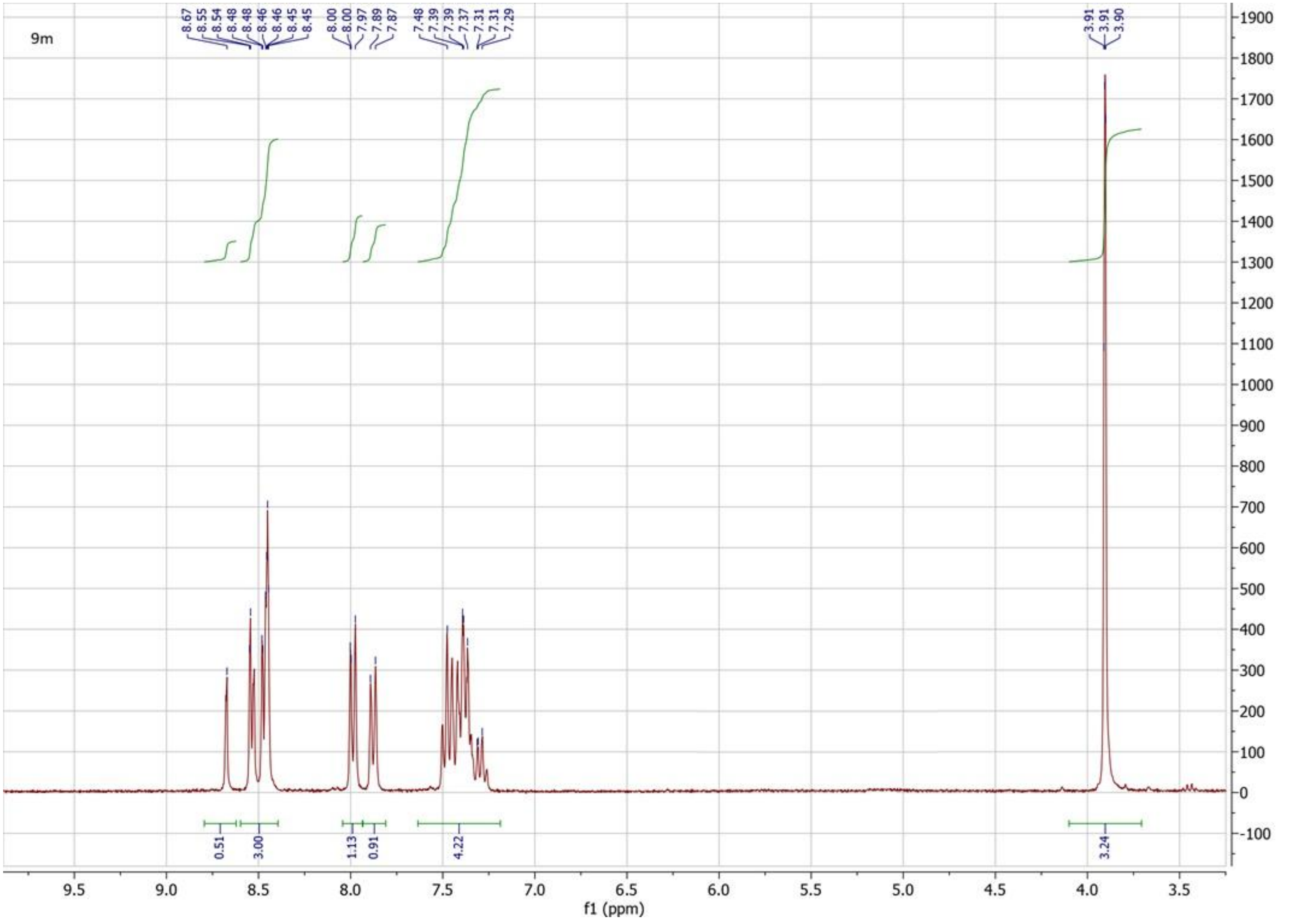
9m
8.67
8.55
8.54
8.48
8.48
8.46
8.46
8.45
8.45
8.00
8.00
7.97
7.89
7.87
7.48
7.39
7.39
7.37
7.31
7.31
7.29
3.91
3.91
3.90
0.51
3.00
1.13
0.91
4.22
3.24
f1 (ppm)

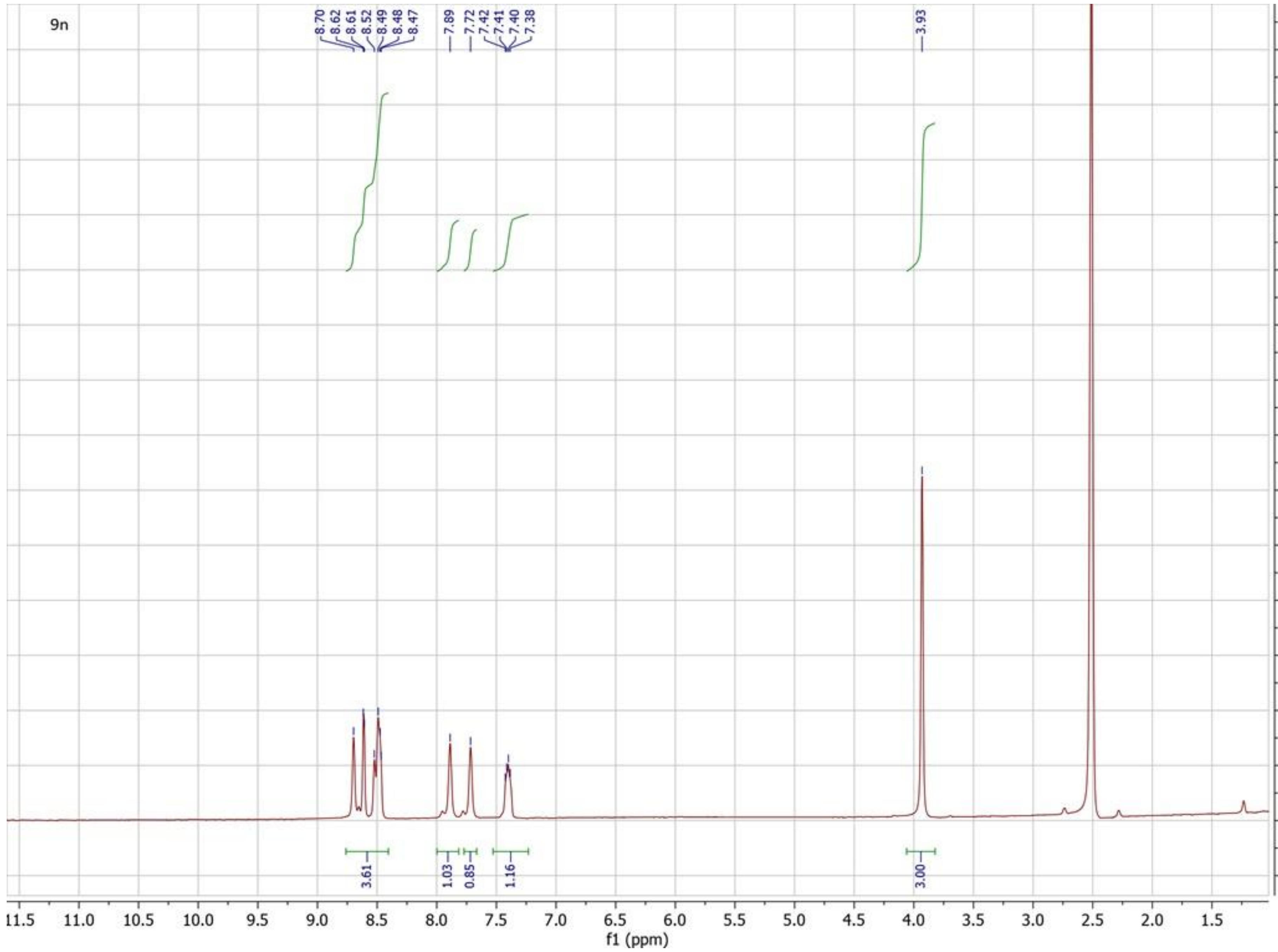
9n
8.70
8.62
8.61
8.52
8.49
8.48
8.47
7.89
7.72
7.42
7.41
7.40
7.38
3.93
3.61
1.03
0.85
1.16
3.00
f1 (ppm)

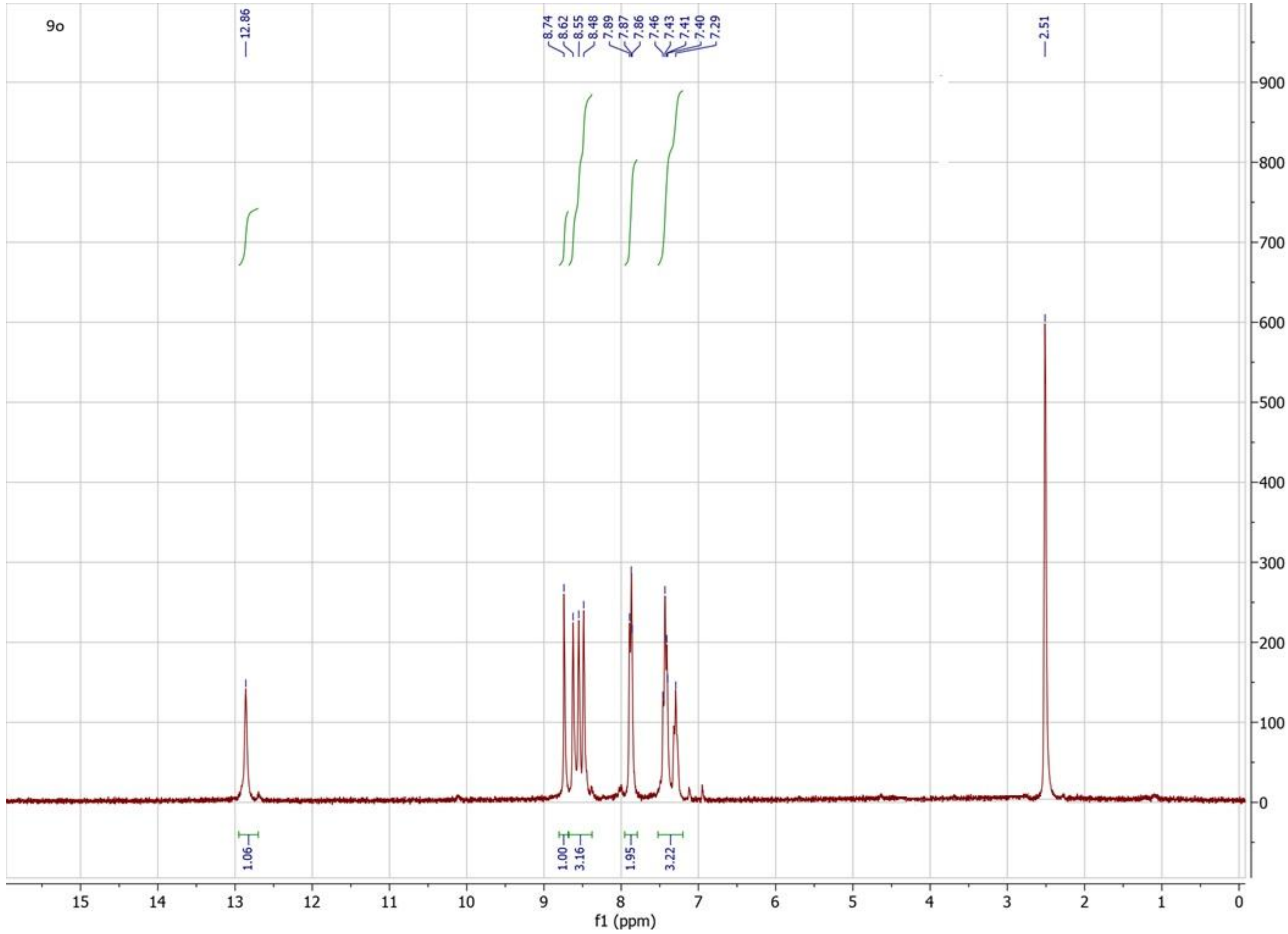
9o
12.86
8.74
8.62
8.55
8.48
7.89
7.87
7.86
7.46
7.43
7.41
7.40
7.29
2.51
1.06
1.00
3.16
1.95
3.22
f1 (ppm)

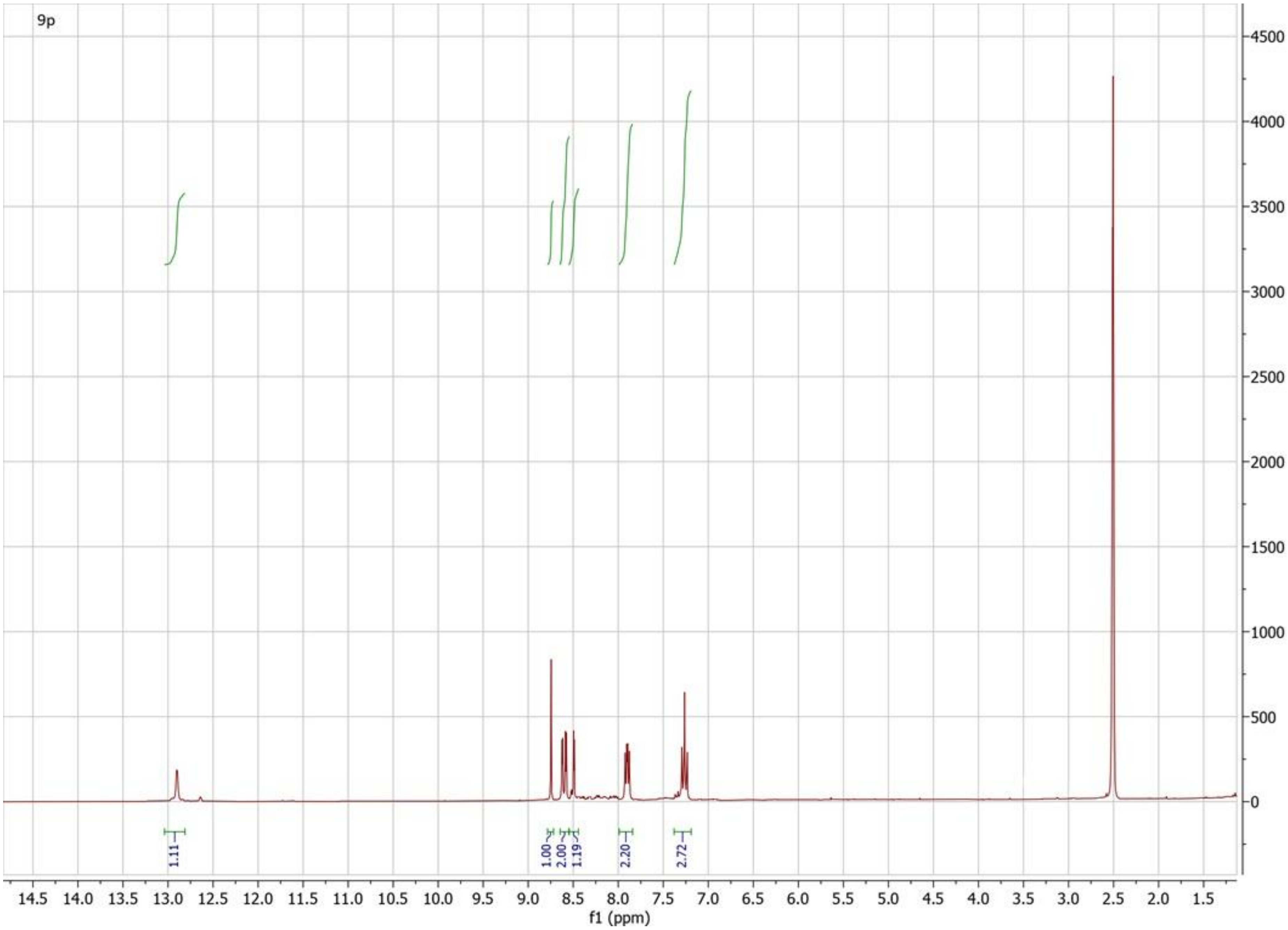
9p
1.11
1.00
2.00
1.19
2.20
2.72
14.5 14.0 13.5 13.0 12.5 12.0 11.5 11.0 10.5 10.0 9.5 9.0 8.5 8.0 7.5 7.0 6.5 6.0 5.5 5.0 4.5 4.0 3.5 3.0 2.5 2.0 1.5
f1 (ppm)
4500 4000 3500 3000 2500 2000 1500 1000 500 0

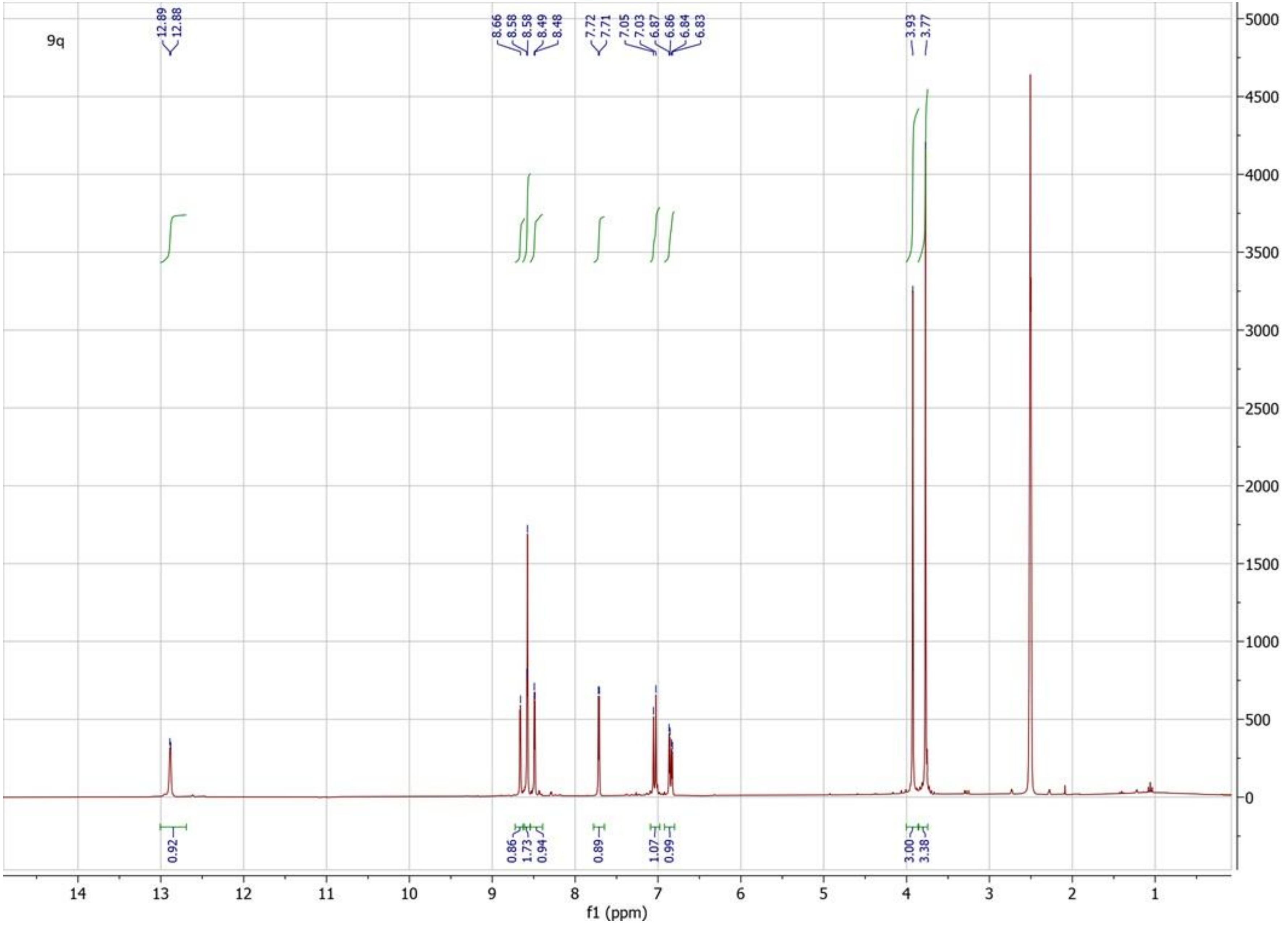
9q
12.89
12.88
8.66
8.58
8.58
8.49
8.48
7.72
7.71
7.05
7.03
6.87
6.86
6.84
6.83
3.93
3.77
0.92
0.86
1.73
0.94
0.89
1.07
0.99
3.00
3.38
f1 (ppm)

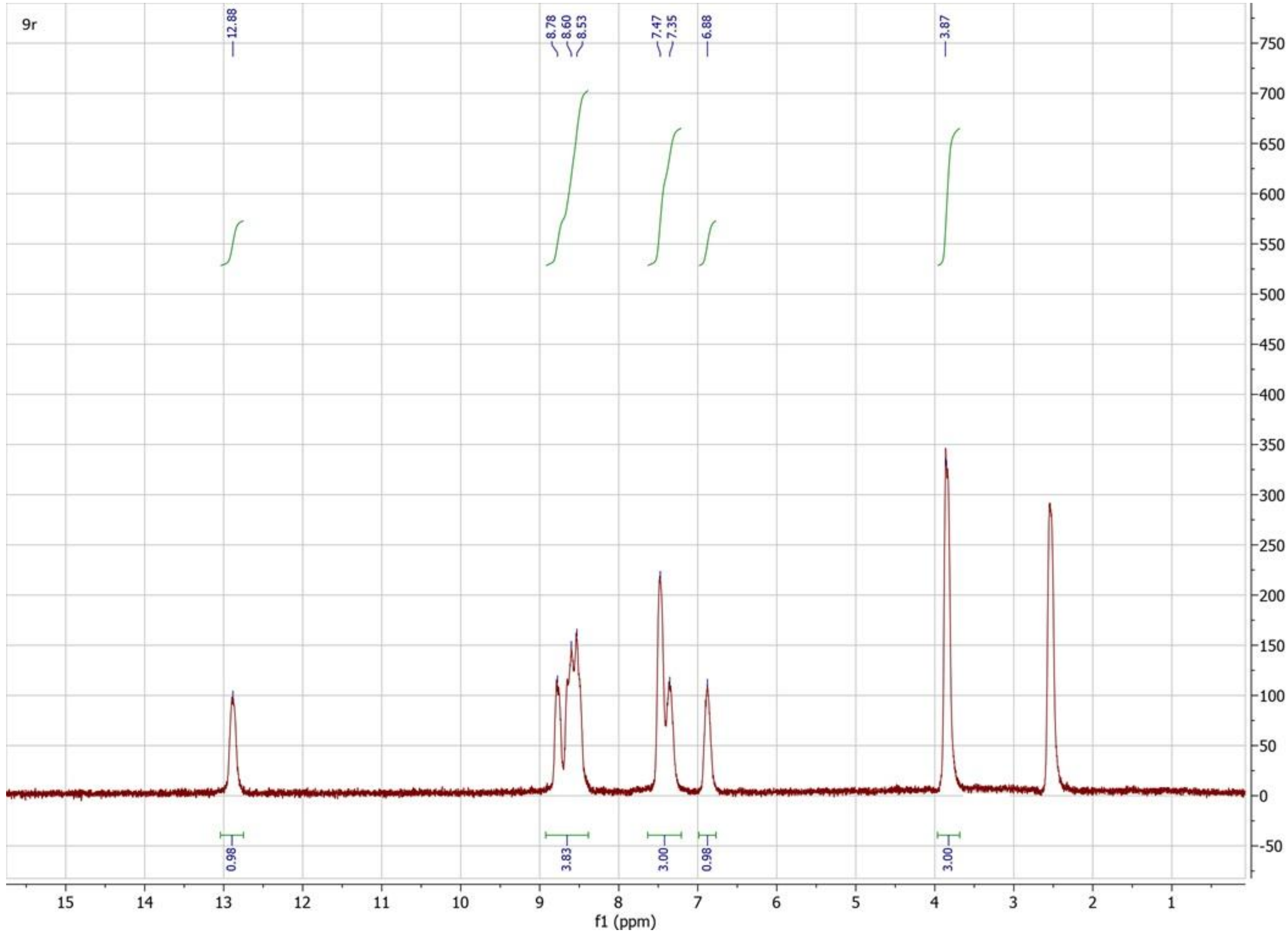
9r
12.88
8.78
8.60
8.53
7.47
7.35
6.88
3.87
0.98
3.83
3.00
0.98
3.00
f1 (ppm)

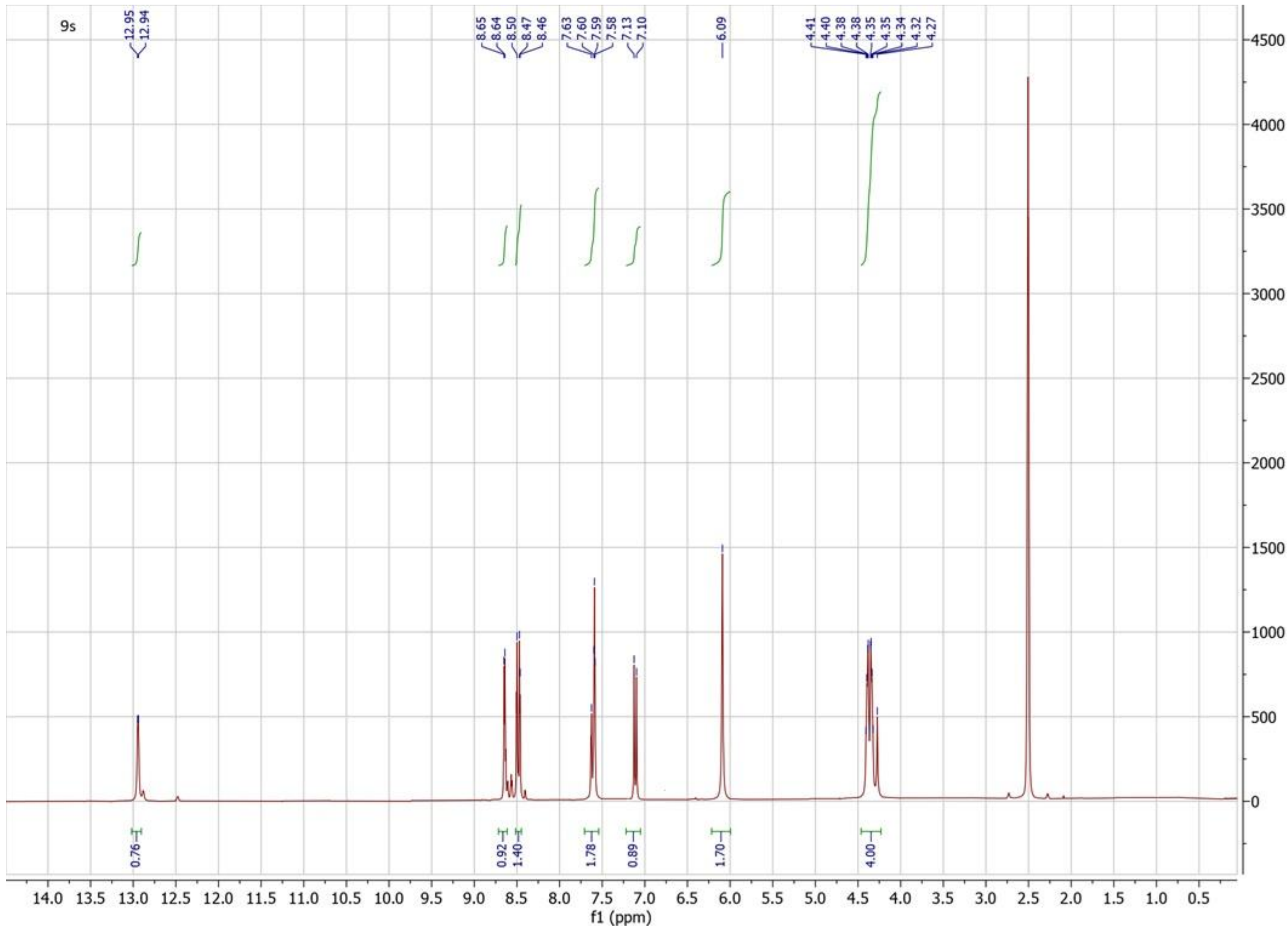
9s
12.95
12.94
8.65
8.64
8.50
8.47
8.46
7.63
7.60
7.59
7.58
7.13
7.10
6.09
4.41
4.40
4.38
4.38
4.35
4.35
4.34
4.32
4.27
0.76
0.92
1.40
1.78
0.89
1.70
4.00
f1 (ppm)

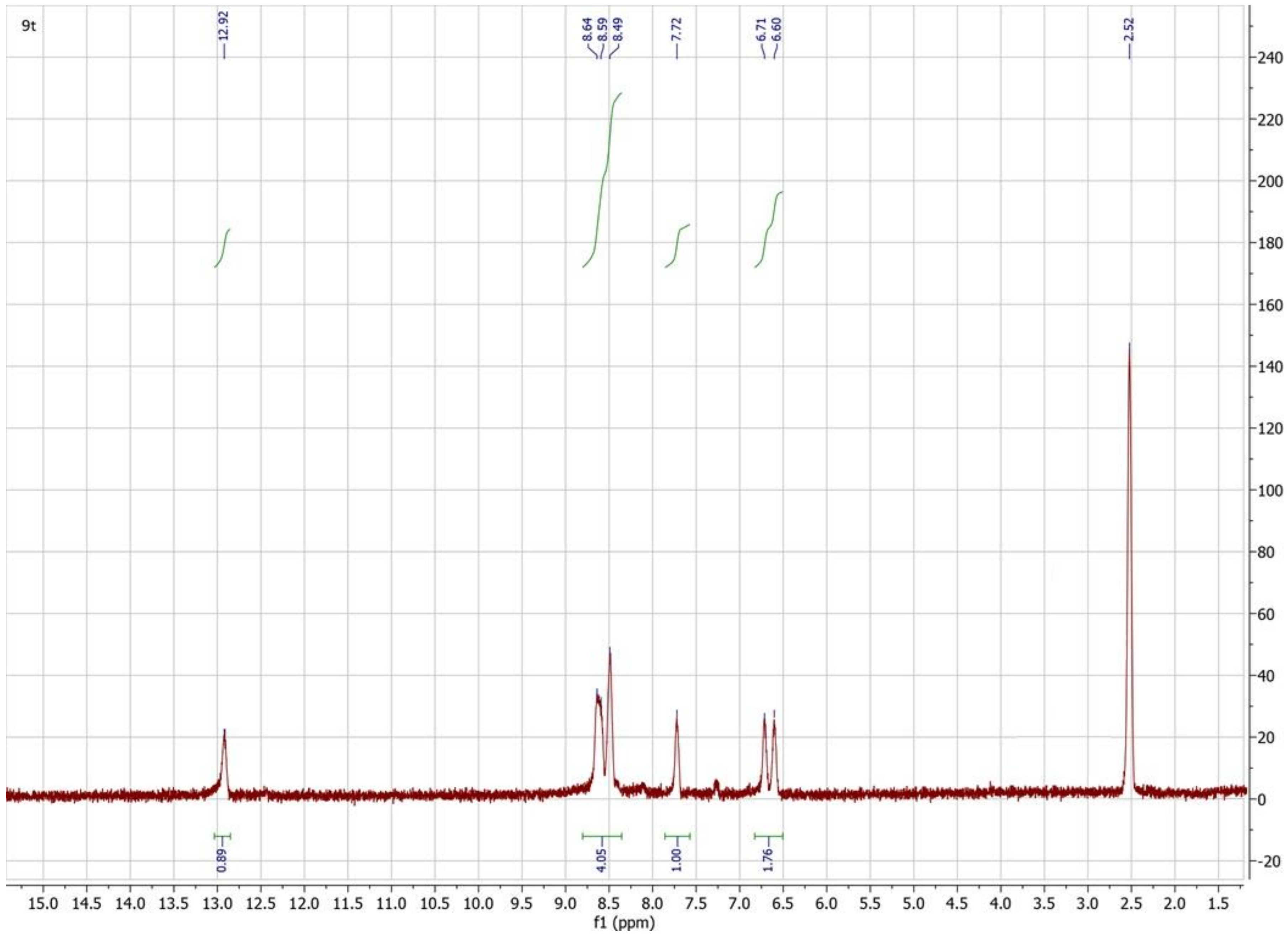
9t
12.92
8.64
8.59
8.49
7.72
6.71
6.60
2.52
0.89
4.05
1.00
1.76
15.0 14.5 14.0 13.5 13.0 12.5 12.0 11.5 11.0 10.5 10.0 9.5 9.0 8.5 8.0 7.5 7.0 6.5 6.0 5.5 5.0 4.5 4.0 3.5 3.0 2.5 2.0 1.5
f1 (ppm)
240 220 200 180 160 140 120 100 80 60 40 20 0 -20

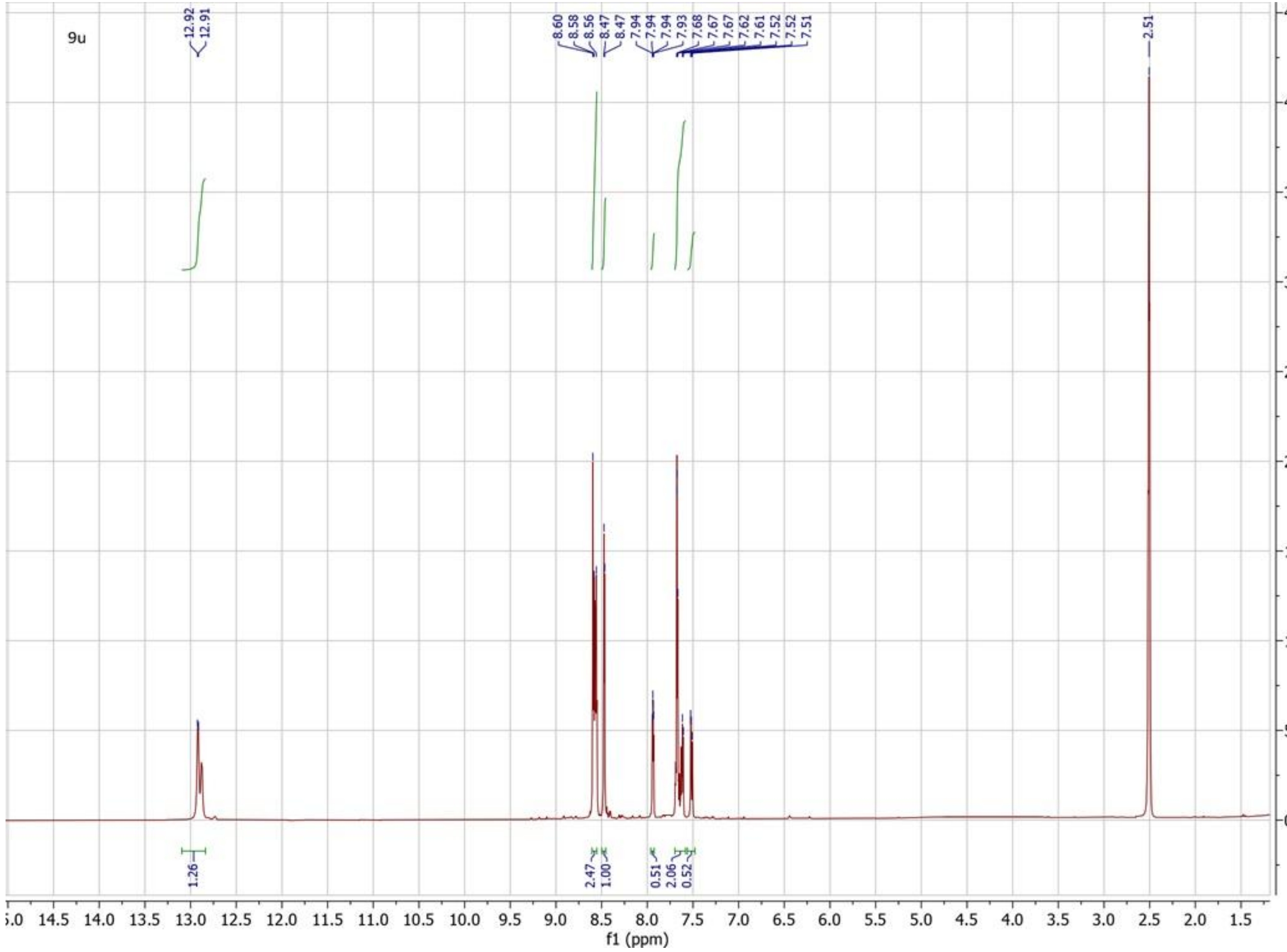
9u
12.92
12.91
8.60
8.58
8.56
8.47
8.47
7.94
7.94
7.94
7.93
7.68
7.67
7.67
7.62
7.61
7.52
7.52
7.51
2.51
1.26
2.47
1.00
0.51
2.06
0.52
f1 (ppm)

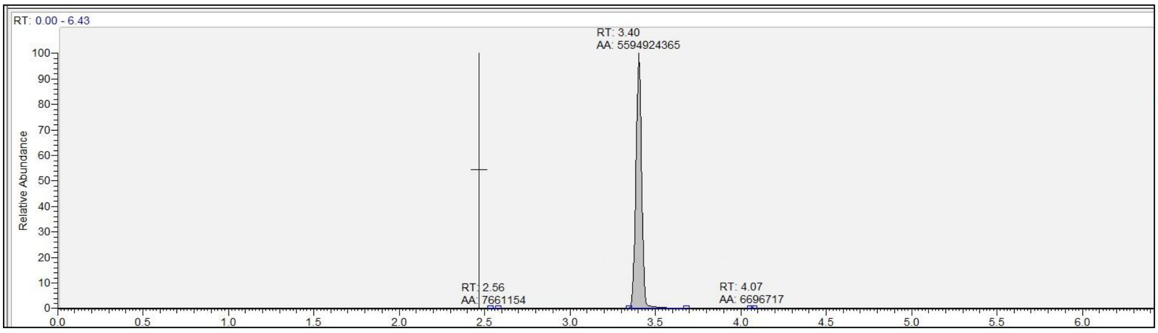

Fig. S1: Compound 9g - $C_{20}H_{17}N_5O_2S$: m/z 392.1175, LC/MS Chromatogram

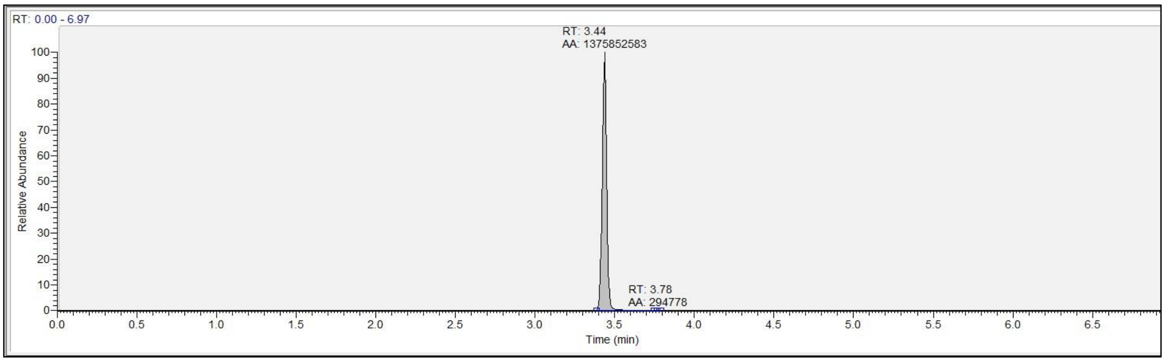

Fig.S2: Compound 9u $C_{15}H_8N_5S_2Br$, m/z 401.9477 LC/MS Chromatogram
